# Adsorption Mechanisms of RNA on Advanced Porous Materials: Current Status and Future Perspectives in Environmental Remediation and Biosafety

**DOI:** 10.3390/ma19214536

**Published:** 2026-10-28

**Authors:** Małgorzata Wasilewska, Maciej Sylwester Bryś, Milena Jaremek, Agata Kobyłka, Natalia Korcz, Paulina Gil-Kulik

**Affiliations:** 1 Department of Physical Chemistry, Institute of Chemical Sciences, Faculty of Chemistry, Maria Curie-Sklodowska University, Maria, Curie-Sklodowska Sq. 3, 20-031 Lublin, Poland; 2 Department of Invertebrate Ecophysiology and Experimental Biology, Faculty of Environmental Biology, University of Life Sciences in Lublin, Doświadczalna 54, 20-280 Lublin, Poland; maciej.brys@up.edu.pl (M.S.B.); milena.jaremek@up.edu.pl (M.J.); 3 Department of Tourism and Recreation, University of Life Sciences in Lublin, Akademicka 15 Street, 20-950 Lublin, Poland; agata.kobylka@up.edu.pl; 4 Department of Geomatics, Forest Research Institute, Sękocin Stary, 3, Braci Leśnej Street, 05-090 Raszyn, Poland; n.korcz@ibles.waw.pl; 5 Department of Clinical Genetics, Medical University of Lublin, 11, Radziwillowska Str., 20-080 Lublin, Poland; pgil.poczt@vp.pl

**Keywords:** RNA adsorption, environmental RNA (eRNA), advanced porous materials, adsorption mechanisms, metal–organic frameworks (MOFs), covalent organic frameworks (COFs), biosafety, environmental remediation

## Abstract

**Highlights:**

- Environmental RNA emerges as a challenge for remediation and biosafety.
- Advanced porous materials provide versatile platforms for RNA capture.
- Adsorption mechanisms differ substantially between ssRNA and dsRNA.
- Environmental factors strongly influence RNA adsorption and desorption.
- Translating laboratory RNA adsorption studies into environmental applications remains a major challenge.

**Abstract:**

Environmental RNA (eRNA), derived from living organisms, pathogens, and double-stranded RNA (dsRNA)-based biopesticides, is becoming increasingly important in the contexts of environmental monitoring, biosafety, and environmental remediation. Although RNA is considered an unstable molecule, its stability can increase through interactions with solid surfaces and porous materials, thereby influencing its transport, accumulation, and bioavailability in the environment. This review summarizes the current state of knowledge regarding RNA adsorption onto advanced porous materials, including metal–organic frameworks (MOFs), covalent organic frameworks (COFs), mesoporous silica, carbon-based materials, biochar, porous polymers, and hybrid materials. It outlines RNA binding mechanisms—such as electrostatic interactions, hydrogen bonding, π–π interactions, and metal-ion complexation—as well as the influence of environmental factors such as pH, ionic strength, and the presence of organic substances. Methods for studying RNA adsorption, adsorbent regeneration and reusability, and potential applications in wastewater treatment, environmental monitoring, and the mitigation of the spread of biologically active RNA are also discussed. This review highlights the persistent gap between laboratory-scale adsorption studies and the real-world deployment of RNA-capturing materials in complex environmental matrices and proposes an integrative framework tracing how RNA type and material properties jointly shape adsorption mechanisms and, in turn, biosafety outcomes.

## 1. Introduction

Ribonucleic acids (RNA) occur in the environment not only as components of living cells but also as extracellular biological material released by organisms and microorganisms into the surrounding environment. RNA can enter the environment through cell lysis and disintegration, as well as via active secretory processes—such as the release of extracellular vesicles containing various classes of RNA [1,2,3]. The presence of such structures has also been confirmed directly in aquatic environments, where aquatic organisms can release extracellular vesicles containing specific RNA molecules that, thanks to encapsulation, may be partially protected against rapid degradation [3]. In a broader sense, environmental RNA (eRNA) refers to RNA obtained from environmental samples—such as water, soil, sediment, or air—and can encompass both RNA associated with cells and other biological structures and its extracellular fractions [4,5].

In recent years, eRNA has gained importance as a biomonitoring tool that complements environmental DNA (eDNA) analysis, as its relatively rapid turnover and the link between specific transcripts and metabolic activity can enhance the temporal resolution of observations and enable the identification of biologically active or recently present organisms [4,5,6]. At the same time, the shorter persistence of RNA compared to DNA should not be regarded as a universal characteristic, as its stability depends on its form and environmental conditions. Recent studies on aquatic systems indicate that extracellular RNA can persist even under disinfection stress, while its association with protective structures, such as extracellular vesicles, may limit the rate of degradation [3,7]. Consequently, the sources, forms of occurrence, persistence, and environmental fate of RNA constitute important issues from the perspectives of both biological monitoring and the assessment of its transport, retention, and potential biological activity in environmental systems.

In the context of monitoring environmental RNA, adsorption offers a promising approach for capturing and immobilizing it on the surfaces of solid materials. Adsorption is widely employed in water treatment technologies due to the relative simplicity of the process design, the availability of a broad range of sorbent materials, and the potential for their regeneration and reuse [8,9]. For RNA, materials characterized by high specific surface area, controlled porosity, and susceptibility to surface chemical modification are of particular importance, as these properties determine the availability of adsorption sites and the nature of the interactions between polyanionic RNA molecules and the sorbent surface. Direct RNA adsorption capability has been demonstrated for materials such as mesoporous silica, with process efficiency depending on particle size, pore diameter, and the type and degree of surface functionalization [10,11,12]. Cho et al. [12] further demonstrated that RNA adsorption onto mesoporous silica can be facilitated by positively charged polyamines, and that electrostatic interactions and hydrogen bonding play a significant role in the binding process.

Carbon-based materials also exhibit a high affinity for RNA binding. Graphene oxide can immobilize single-stranded RNA (ssRNA)—for instance, through π–π interactions between nucleic acid bases and the graphene surface—and surface binding can further inhibit enzymatic degradation of the RNA [13]. Research on biochar is particularly significant from an environmental perspective, having demonstrated the removal of over 90% of RNA and DNA fragments from wastewater; this highlights the potential of low-cost carbon materials to mitigate the presence of extracellular nucleic acids in aquatic systems [14].

At the same time, the development of materials with engineered porous structures, such as covalent organic frameworks (COFs), offers the possibility of more controlled RNA binding through the appropriate tuning of charge, surface chemistry, and pore architecture. For instance, cationic COFs demonstrate effective siRNA adsorption, whereas guanidinium-based COFs enable reversible RNA binding and stabilization via non-covalent interactions between the material framework and nucleic acid bases [15,16]. Current findings thus indicate that RNA adsorption on porous materials can be governed by a combination of electrostatic interactions, hydrogen bonding, π–π interactions, and effects related to porosity and surface chemistry; however, the potential of these mechanisms for removing RNA from real-world environmental matrices remains relatively poorly understood.

Interest in environmental RNA and RNA adsorption has grown considerably in recent years, driven by advances in wastewater-based epidemiology, environmental surveillance, nucleic acid recovery technologies, and RNA-based therapeutics. The COVID-19 pandemic further highlighted the importance of understanding the environmental fate, persistence, and capture of RNA species in complex matrices. Consequently, RNA interactions with porous materials have attracted growing attention across environmental science, materials engineering, biotechnology, and biosafety research.

**Figure 1:**
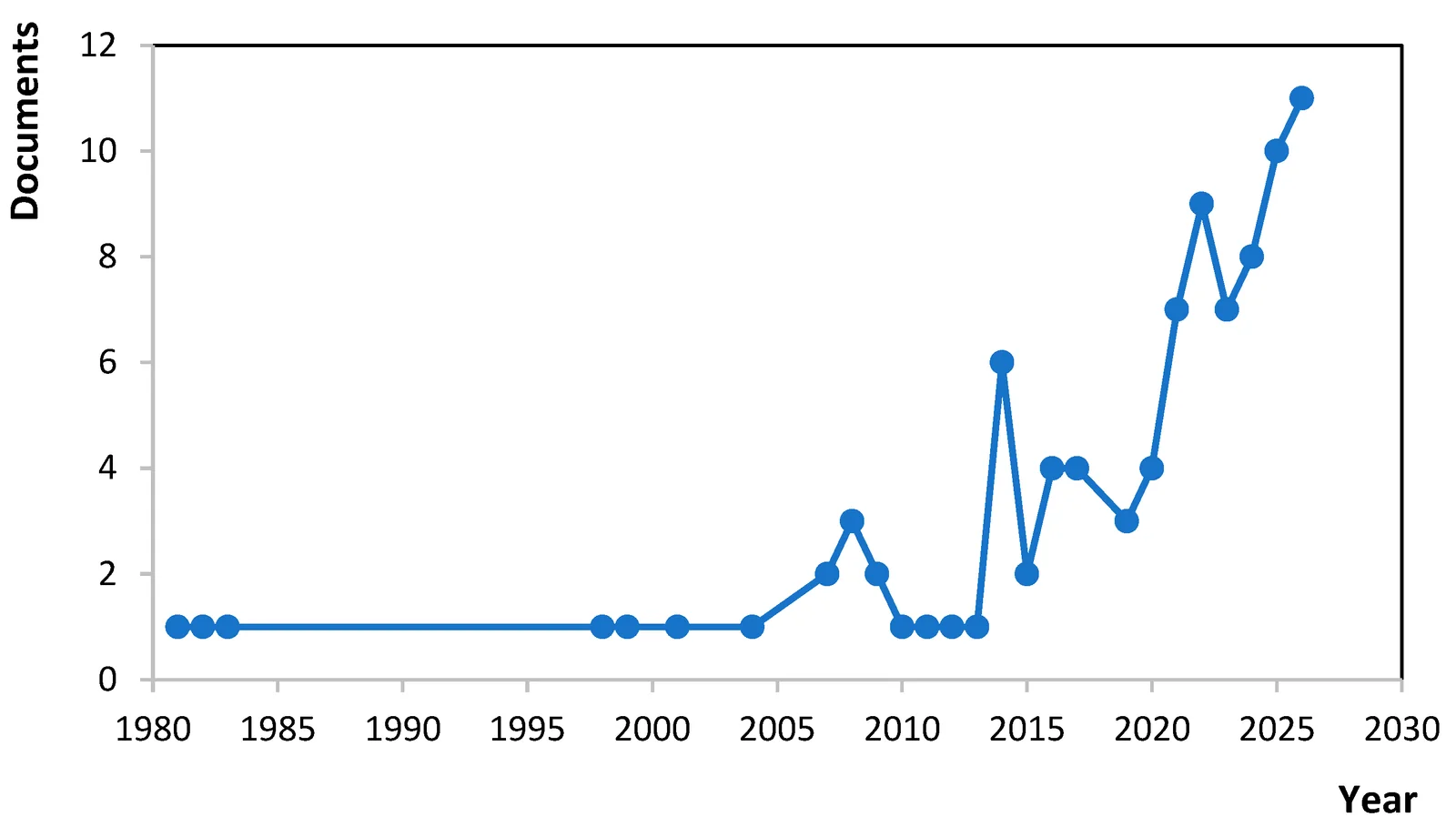
Publication trend for research related to RNA adsorption and porous materials based on a Scopus search using the keywords “RNA”, “adsorption”, and “porous” (search performed in October 2026).

This growing interest is reflected in publication trends. A Scopus search performed using the keywords “RNA”, “adsorption”, and “porous” identified 93 relevant documents (Figure 1). The number of publications increased from 4 in 2020 to 11 in 2026, highlighting the rapidly expanding interest in RNA adsorption, advanced porous materials, environmental RNA monitoring, wastewater-based epidemiology, and RNA-based technologies. The most pronounced growth has occurred during the last five years, reflecting the increasing importance of environmental surveillance and RNA-based applications.

Despite this growing interest, existing review articles have typically focused on nucleic acid adsorption [17,18], RNA immobilization [19,20], porous materials [21,22], or environmental monitoring [6,23,24] separately. A comprehensive analysis integrating RNA physicochemical properties, adsorption mechanisms, advanced porous adsorbents, environmental matrices, and biosafety considerations remains lacking.

Nevertheless, current knowledge in this area remains fragmented and scattered across research in materials science, biotechnology, and environmental science. Existing studies indicate that RNA adsorption efficiency is closely linked to the physicochemical properties of the adsorbent, such as specific surface area, pore size and accessibility, surface charge, and the type and density of functional groups [11,12]. At the same time, there is a lack of systematic research to unequivocally establish the relationships between material structure and chemistry and the adsorption of various RNA forms—specifically ssRNA, dsRNA, and short RNA fragments. The stability of adsorbed RNA also remains an important yet poorly understood issue. It has been shown that changes in chemical conditions can lead to desorption; for instance, RNA bound to mesoporous silica can be released again in the presence of salts, EDTA, or urea due to the weakening of electrostatic interactions and hydrogen bonds [12]. Consequently, evaluating the suitability of adsorbent materials for environmental applications requires considering not only adsorption capacity and kinetics but also the durability of immobilization and the risk of secondary release of biologically active RNA.

A particularly significant research gap concerns the translation of findings obtained in controlled laboratory systems to real-world environmental matrices—such as surface waters, wastewater, or agricultural runoff—where factors such as pH, ionic strength, dissolved organic matter, proteins, colloids, extracellular DNA, microplastics, other biomacromolecules, and nuclease activity can significantly alter adsorption and desorption processes through competitive adsorption and changes in surface interactions. Although the effective removal of RNA has already been demonstrated using materials such as biochar in wastewater [14], the number of studies evaluating the behavior of various RNA forms on advanced porous materials under actual environmental conditions remains limited. At the same time, research on eRNA highlights the need for further optimization and standardization of procedures, as well as an increase in field studies to enable the reliable translation of laboratory findings to environmental systems [5]. The potential of engineered materials—such as MOFs and COFs—remains largely underexplored; while these materials are being intensively developed for applications involving RNA immobilization, protection, and delivery, their use for the controlled capture of RNA from water and wastewater and the mitigation of its environmental bioavailability has not been sufficiently investigated. Consequently, there is a lack of a coherent framework linking material structural properties, RNA type, adsorption mechanisms, environmental conditions, and the stability of the resulting material–RNA complex. Bridging this gap is crucial for the rational design of materials capable of the selective and durable capture of RNA, as well as for assessing their potential practical application in environmental remediation and biosafety systems. In complex environmental matrices, competition for adsorption sites may substantially affect RNA removal efficiency. Various constituents of natural waters and wastewater, including dissolved organic matter, proteins, extracellular DNA, colloids, and microplastics, may interfere with RNA adsorption by blocking active sites, modifying surface charge, altering pore accessibility, or participating in competitive adsorption processes. Therefore, adsorption data obtained in simplified laboratory systems should be translated to environmental applications with caution.

In contrast to earlier reviews that have largely treated adsorbent design, RNA immobilization, environmental RNA monitoring, and RNA biology as separate lines of inquiry, this review provides a critical and integrated assessment of RNA adsorption on advanced porous materials. To the best of our knowledge, no previous review has comprehensively integrated RNA physicochemical properties, adsorption mechanisms, advanced porous adsorbents, environmental matrices, and biosafety considerations within a single structure–property–performance framework. Particular attention is paid to the influence of specific surface area, pore structure and size, surface charge, and surface functionalization on the adsorption of various RNA forms—including ssRNA and dsRNA—as well as the roles of electrostatic interactions, hydrogen bonding, hydrophobic interactions, and π–π interactions. Major classes of porous materials—specifically MOFs, COFs, mesoporous silica materials, carbon-based materials, biochar, and porous polymers—are analyzed with regard to their potential for RNA capture and immobilization. Kinetic and equilibrium models used to describe the adsorption process are evaluated, alongside the impact of environmental conditions (such as pH, ionic strength, temperature, and the presence of competing species) on adsorption efficiency, the stability of material–RNA complexes, and the potential for RNA desorption. Based on this analysis, the feasibility of translating results obtained under controlled laboratory conditions to real-world environmental systems is critically assessed, with a specific focus on water and wastewater treatment, agricultural runoff management, and the control of environmental RNA relevant to biosafety. Furthermore, key methodological and technological limitations are identified, as are directions for future research aimed at developing selective, stable, and regenerable adsorbent materials for RNA control in environmental applications.

## 2. Environmental and Biosafety-Relevant RNA: Characteristics and Behavior

### 2.1. Types and Sources of Environmental RNA

RNA in the environment constitutes a diverse group of biomolecules derived from living and dead cells, extracellular material, extracellular vesicles, and viral particles. In recent years, environmental RNA has gained particular importance in molecular ecology, biodiversity monitoring, environmental epidemiology, biological risk assessment and pathogen surveillance. Compared to environmental DNA, RNA is typically more susceptible to degradation, potentially providing information about relatively recent organismal activity. However, this does not mean that all types of RNA have an equally short survival time. The stability of molecules depends on their type, length, secondary structure, mode of release into the environment, protection by membranes or proteins, as well as temperature, pH, the presence of enzymes, metal ions, dissolved organic matter, and sorption surfaces [25,26,27]. Mitochondrial mRNA was the least stable component studied and disappeared within several hours, whereas ribosomal eRNA persisted longer, although it still degraded faster than the corresponding eDNA. These results indicate that the concept of “RNA stability” should not be considered as a single property of an entire group of molecules, but as a feature dependent on both the RNA type and the environmental conditions [27].

In the environment, cellular, extracellular, and viral RNAs can be distinguished. Cellular RNA includes mRNA, rRNA, tRNA, and regulatory RNAs found in bacteria, archaea, fungi, algae, plants, animals, and other organisms. Following cell death or damage, this material can enter the extracellular pool. Extracellular RNA can exist as a free molecule in the aqueous phase, be bound to proteins or other macromolecules, adsorb to minerals and colloids, or be contained within extracellular vesicles. Luz et al. [28] demonstrated that *Staphylococcus aureus* extracellular vesicles contain numerous types of RNA, including mRNA and small regulatory RNAs, and their composition depends on environmental conditions and the bacterial growth phase. Transcripts associated with virulence were also detected among the vesicle-associated RNAs. These results show that extracellular RNA does not have to be just a random product of cell decay, but may also be transported in biological structures that potentially protect it from direct environmental impact and participate in intercellular communication [28].

Viral RNA is particularly important from a biosafety perspective. It can be found within an infectious virion, within a damaged viral particle that has lost its infectivity, or present as free or partially degraded genetic material. Therefore, detecting RNA by RT-qPCR does not confirm the presence of an infectious virus. Distinguishing between nucleic acid persistence and persistence of infectivity is fundamental to the interpretation of environmental data. Korajkic et al. [29] directly compared the decay rate of infectious SARS-CoV-2 and its RNA in sewage and environmental waters. These studies confirmed that the parameters describing loss of infectivity and loss of detectable genetic signal are not equivalent and depend on temperature and matrix type. This means that the presence of viral RNA can be an excellent epidemiological indicator, but it does not, in itself, constitute evidence of the virus’s transmissibility [29].

A similar conclusion was drawn from studies using viral surrogate models. Corimayo et al. [30] analyzed bacteriophages P22 and PP7, representing DNA and RNA virus models, respectively, in synthetic aqueous matrices with different temperatures, electrical conductivity, and sediment content. Both infectivity and nucleic acid detectability were clearly dependent on environmental conditions, and both phages persisted longer at 12 °C than at 25 °C. The presence of sediment particles reduced their persistence, while the effect of conductivity was dependent on the virus type. Importantly, the relative persistence of infectivity and nucleic acid differed between the two models, further demonstrating that a single universal conversion factor cannot be used to translate a molecular signal into potential infectious risk [30].

### 2.2. Physicochemical Properties of RNA Relevant to Adsorption

One of the fundamental chemical properties of RNA that determines its behavior in the environment is the negative charge of its phosphodiester backbone. Under conditions close to neutral pH, RNA functions as a polyanion; therefore, its adsorption to mineral and colloidal surfaces is largely controlled by electrostatic interactions. The negative charge may limit direct binding to negatively charged surfaces, but the presence of cations, particularly Ca^2+^ and Mg^2+^, allows for charge shielding or the formation of ionic bridges. Furthermore, the structure of RNA and the presence of the 2′-OH group of ribose contribute to its greater chemical susceptibility to hydrolysis than DNA. In an alkaline environment, the 2′-OH group can participate in intramolecular nucleophilic attack, leading to cleavage of the phosphodiester bond. Studies by Li et al. [31], conducted on mRNA and miRNA in biological samples subjected to different environmental conditions, also showed that different RNA types exhibit different levels of stability. mRNA was more susceptible to high temperature and humidity than miRNAs, further indicating that the size and structure of the molecule are important determinants of its stability.

Beyond charge characteristics, several physicochemical properties collectively govern RNA adsorption on porous materials, including molecular dimensions, conformational flexibility, secondary and tertiary structures, and the accessibility of functional groups involved in surface interactions. These features influence pore accessibility, steric effects, electrostatic attraction, hydrogen bonding, cation bridging, and interactions with surface functional groups.

Importantly, different RNA species, including ssRNA, dsRNA, mRNA, miRNA, viral RNA, and extracellular RNA, and formulated or encapsulated RNA, may exhibit substantially different adsorption behaviours owing to variations in size, conformation, structural complexity, surface accessibility, and environmental stability. Consequently, adsorption mechanisms and efficiencies reported for one RNA type cannot always be directly generalized to other RNA classes, highlighting the need for RNA-type-specific evaluation of adsorbent performance.

Collectively, the polyanionic nature of RNA, the presence of the ribose 2′-OH group, molecular size, strand organization, conformational flexibility, and secondary structure govern its interactions with environmental surfaces. These properties influence electrostatic attraction, hydrogen bonding, cation bridging, pore accessibility, and other adsorption processes occurring at mineral and engineered material interfaces. Consequently, the physicochemical characteristics of RNA play a central role in determining both sorption efficiency and environmental persistence.

The physicochemical characteristics of RNA discussed above directly determine its adsorption behaviour on porous materials. Molecular charge, size, conformational flexibility, and secondary structure influence the relative contributions of electrostatic attraction, hydrogen bonding, cation bridging, π–π interactions, and steric effects. Therefore, understanding RNA structure is essential for elucidating the adsorption mechanisms governing its interaction with mineral and engineered surfaces.

### 2.3. Stability and Degradation of RNA in the Context of Biosafety

The effect of pH on eRNA was analyzed in detail by Kagzi et al. [25]. The authors compared the degradation of eDNA and *Daphnia pulex* eRNA over a wide pH range and showed that eRNA decayed faster than eDNA under all conditions analyzed. The eRNA detectability at neutral pH was up to approximately 57 h, whereas eDNA, especially under acidic conditions, could remain detectable much longer. The results confirm the usefulness of eRNA as a marker with high temporal resolution, but at the same time demonstrate that interpreting its presence requires knowledge of the physicochemical properties of the specific environment [25]. The more recent studies by Brandão-Dias et al. [27] expanded on this concept by demonstrating a biphasic degradation pattern for different forms of environmental nucleic acids (eNA): an initial rapid loss of material was followed by a slower decay phase. This indicates that simple single-phase degradation models may not always be sufficient to estimate the time elapsed since the material was released into the environment.

Metal ions also play an important role in RNA stability. Chatterjee et al. [32] demonstrated that some metal cations can catalyze RNA hydrolysis in aqueous environments even at ambient temperature and near-neutral pH. A particularly significant effect was observed for stronger Lewis acids, such as Cu^2+^ and Pb^2+^. Single-stranded RNA was more susceptible to this reaction than double-stranded RNA, indicating that secondary structure may limit metal access to vulnerable sites in the phosphodiester backbone. At the same time, metal-complexing ligands can reduce their catalytic activity. The importance of metal ions is therefore twofold: they can enhance RNA sorption on certain surfaces by shielding the negative charge, while simultaneously accelerating its chemical degradation under certain conditions [32].

These findings demonstrate that RNA degradation is governed by multiple interacting environmental factors and cannot be described using a single universal decay model. Understanding these processes is essential for interpreting environmental RNA signals and assessing their relevance to biological risk.

### 2.4. Interactions of RNA with Aquatic Matrices and Their Implications for Remediation and Biosafety

The complexity of the interactions between adsorption and degradation becomes even more apparent at the mineral–water interface. Zhang et al. [33] showed that RNA can undergo rapid, abiotic hydrolysis upon binding to iron oxides and oxyhydroxides, particularly goethite. The process occurred on an hourly time scale, with surface iron centers acting as Lewis acids catalyzing the sequence-nonspecific cleavage of phosphodiester bonds. These results are important for modeling the environmental fate of RNA because they challenge the simple assumption that adsorption to a mineral surface always protects a nucleic acid from degradation. Depending on the mineralogical nature of the surface, sorption can stabilize the molecule or, conversely, increase the rate of its degradation [33].

However, binding RNA to environmental components can increase its persistence. Chatterjee et al. [34] demonstrated that dissolved organic matter (DOM), including humic substances, can form complexes with RNA and limit its accessibility to RNases. This protective effect was observed both in model solutions containing humic and fulvic acids and in natural matrices. This indicates that DOM can prolong the detection time of RNA in water despite the presence of active degradative enzymes [34]. This phenomenon is important for monitoring, as samples with similar temperature, pH, and initial RNA concentration can exhibit different decay rates depending on the organic matter. This also means that the genetic material of pathogens can remain molecularly detectable after losing biological activity if effectively protected by matrix components.

Microplastics are also important contemporary environmental surfaces. Maguire et al. [35] demonstrated that free extracellular nucleic acids can adsorb to microplastics in natural and synthetic waters. However, the intensity of the process depended on the matrix; adsorption was observed particularly in freshwater and stormwater, whereas in wastewater it was limited, likely due to competition with biomacromolecules and nuclease activity. The authors suggest that microplastics may represent an additional carrier of environmental genetic material and potentially influence its transport, spatial distribution, and accessibility to degradation processes [35]. Forest ecosystems represent another important environmental compartment where adsorption onto soil particles, organic matter, and natural porous matrices may influence the persistence, mobility, and environmental fate of RNA.

This issue is particularly relevant in the case of environmental release of dsRNA used in RNA interference technologies. Parker et al. [36] demonstrated that dsRNA used as a potential RNAi pesticide in the soil is simultaneously subject to adsorption on soil particle surfaces, degradation in solution, and potential uptake by microorganisms. This shows that the environmental safety assessment of RNA-based products should take into account not only the chemical half-life of the molecule, but also its distribution between the aqueous and solid phases, as well as the possibility of biological transformation [36].

An additional factor influencing RNA stability is encapsulation. Zarrabian and Sherif [37] compared naked and encapsulated dsRNA in water and on plant surfaces. Encapsulation in minicells significantly increased the stability of dsRNA, and the half-life was more than twice as long as that of the unprotected molecule. The stability was modified by water hardness, including the presence of Ca^2+^ and the activity of microorganisms. These results have direct implications for environmental safety assessment, as they demonstrate that the formulation and the form of RNA can be as important as the molecule’s sequence. With respect to biosafety, this necessitates separate determination of the naked, protected, and structurally encapsulated RNA [37].

The aqueous matrix is equally important in the monitoring of viral RNA. Recent research by Moharrery et al. [38] on hepatitis C virus (HCV) RNA in wastewater has shown that the matrix composition may have a greater impact on decay kinetics than temperature. The fastest degradation was observed in wastewater, while RNA decayed more slowly in autoclaved and filtered samples, indicating a significant contribution of microbial and enzymatic activity. Higher temperature increased degradation, but this effect was secondary to matrix properties. The authors emphasized that wastewater-based HCV monitoring should incorporate high sampling frequency and models that correct the observed signal for RNA decay processes [38]. These results confirm that interpreting RNA copy number without considering transport time and environmental conditions can lead to an erroneous assessment of the pathogen’s prevalence.

The reliability of the monitoring is also affected by the conditions under which the sample is handled after it has been collected. Williams et al. [39] analyzed the stability of viral RNA and DNA in wastewater as a function of time, storage temperature, and the number of freeze-thaw cycles. In the case of SARS-CoV-2, different regions of the genome did not exhibit identical stability. The authors also showed that, for wastewater samples, storage at 4 °C ensured good recovery of SARS-CoV-2, while repeated freezing and thawing also negatively affected sequencing parameters. At the same time, historical extracts stored at −80 °C for 12–16 months maintained high stability. These results demonstrate that collection, transport, storage, and archiving procedures are integral to the quality of viral RNA monitoring [39].

From a biosafety perspective, the most important principle remains the distinction between detectability of genetic material and infectivity. Viral RNA may remain detectable after the virus has lost its ability to infect the host, and the extent of this discrepancy depends on the virus, temperature, matrix, the presence of sediments and other physicochemical factors [29,30]. Consequently, the RT-qPCR result should be interpreted as information about a specific genomic marker rather than a direct measure of infectious dose. Assessing the actual risk of transmission requires additional information regarding the integrity of the viral particle, its infectivity, dose, route of exposure, and the characteristics of the specific pathogen. On the other hand, wastewater samples cannot be considered safe simply because the RNA marker tested may originate from an inactive virus. The sewage matrix is a complex biological matrix containing many potential pathogens; therefore, sample collection, transport and processing should remain part of the laboratory biological risk assessment.

**Figure 2:**
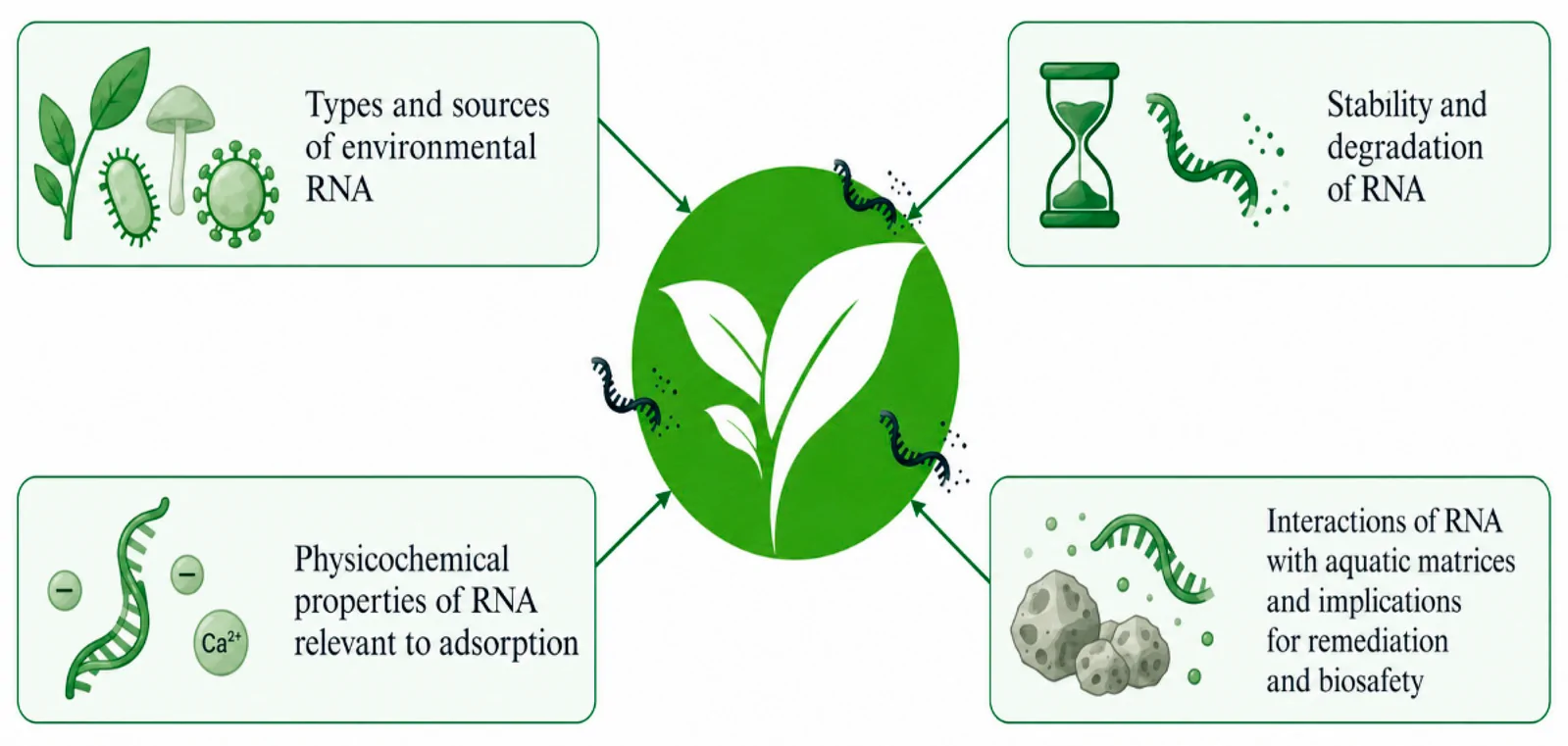
Conceptual overview of environmental RNA sources, properties, stability, and environmental interactions.

The totality of available data indicates that RNA in the environment cannot be described by a single universal half-life. Cellular RNA, RNA present in extracellular vesicles, eRNA, viral RNA, and synthetic or technologically used dsRNA differ in structure, scale of protection, and mode of interaction with the matrix. The stability is controlled simultaneously by temperature, pH, the presence of RNases and microorganisms, metal ions, DOM, minerals, sediments, colloids, microplastics, and the physical form of the molecule. Studies by Kagzi et al. [25] and Brandão-Dias et al. [27] show that eRNA can provide information faster than eDNA, but its different components degrade at different rates. Chatterjee et al. [32] and Zhang et al. [33] demonstrate the importance of chemical and surface-catalyzed hydrolysis, while Chatterjee et al. [34] demonstrate that DOM can have a protective effect. Parker et al. [36], Zarrabian and Sherif [37], and Maguire et al. [35] extend this perspective to technologically applied dsRNA, encapsulation, and interactions with microplastics. Korajkic et al. [29], Williams et al. [39], Corimayo et al. [30], and Moharrery et al. [38] demonstrate that in viral monitoring, matrix conditions and sample handling are crucial for distinguishing between the actual presence of the pathogen and the observed RNA signal. A proper understanding of these processes is essential for the development of reliable environmental monitoring, wastewater-based epidemiology, remediation technologies and biosafety assessment. In practice, it is particularly important to separate three distinct endpoints: removal of genetic material from the aqueous phase, the degradation or loss of RNA integrity, and inactivation of the infectious pathogen. Only by considering these parameters simultaneously is it possible to correctly interpret the environmental presence of RNA and realistically assess its significance for public health and biosafety. The major topics discussed in this section and their relationships to environmental RNA behaviour are summarized schematically in Figure 2.

## 3. Advanced Porous Materials for RNA Adsorption

As discussed in the previous section, the physicochemical properties of RNA, including its polyanionic character, structural organization, and conformational flexibility, strongly influence the mechanisms by which RNA interacts with porous adsorbents. RNA is increasingly utilized in vaccines, therapies, diagnostics, and nucleic acid delivery systems; however, its application is limited by susceptibility to enzymatic degradation and challenges in achieving high purity and stability. Of particular importance is mRNA production via in vitro transcription, a process in which the formation of dsRNA impurities can impair translation and trigger an interferon response [40]. Consequently, advanced porous adsorbents are being intensively developed; their high specific surface area, controlled pore size, and tailored functional group chemistry can enable the control of binding processes, selective separation, and—in suitably designed systems—the mild elution of RNA [22]. However, adsorption performance should not be attributed solely to specific surface area, as accessibility of adsorption sites, surface heterogeneity, molecular dimensions of RNA, and adsorbate–surface interactions may be equally important factors governing adsorption efficiency. Therefore, correlations between RNA adsorption capacity and reported specific surface area should be interpreted with caution.

Specific surface area (SSA) is most commonly determined using the Brunauer–Emmett–Teller (BET) method and is frequently used to characterize porous adsorbents. Nevertheless, BET-derived SSA should not necessarily be regarded as a direct measure of the interfacial area accessible to large biomolecules such as RNA. In heterogeneous porous materials, adsorption behaviour may additionally depend on pore accessibility, hydration effects, surface heterogeneity, and lateral interactions within the adsorbed phase. Recent thermodynamic approaches based on two-dimensional descriptions of adsorption have further highlighted limitations of conventional BET analysis and suggested that the thermodynamically accessible interfacial area may differ from the experimentally reported SSA. Consequently, SSA should be interpreted together with complementary structural and surface characteristics when evaluating RNA adsorption performance [41].

### 3.1. Carbon Materials

Carbon materials include graphene, graphene oxide (GO), reduced graphene oxide, carbon nanotubes, activated carbon, and mesoporous nanocarbons, as well as their magnetic and composite variants [22]. Their unique sorption properties stem from a high specific surface area, the presence of conjugated aromatic systems, the ability to precisely engineer micro- and mesoporous structures, and their susceptibility to functionalization with oxygen, amino, or polymer groups [40]. In this regard, graphene oxide is a particularly promising platform, as it combines an sp^2^-hybridized planar surface with numerous hydrophilic functional groups—specifically hydroxyl, epoxy, and carboxyl groups [40].

**Figure 3:**
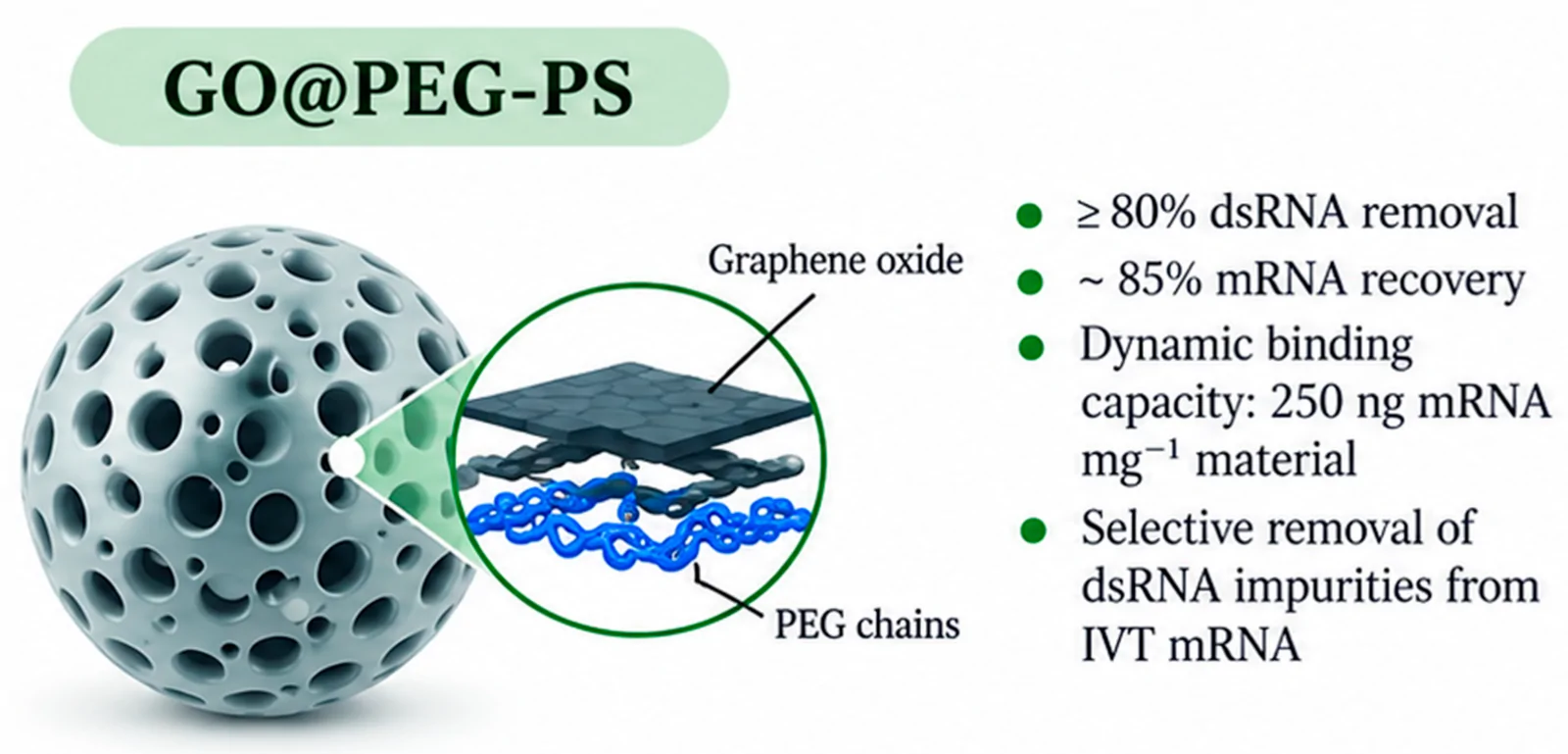
GO@PEG-PS system for selective dsRNA removal from IVT mRNA preparations.

This contrasts with MOF-type structures, where adsorption properties can be tailored through factors such as interactions with metal nodes and the tuning of pore size and geometry [42]. Key mechanisms governing the interaction between graphene oxide and nucleic acids include π–π stacking interactions between the GO plane and nitrogenous bases, hydrogen bonding involving oxygen-containing groups, and electrostatic interactions modulated by the presence of cations such as Mg^2+^, the significance of which depends on, among other factors, on pH and buffer composition [40]. The interplay of these mechanisms can lead to differences in GO affinity for RNAs with varying structures, enabling selective differentiation between ssRNA and dsRNA in the presence of specific divalent cations. This property was successfully utilized in the GO@PEG-PS system (a GO composite on PEGylated polystyrene resin), where the preferential adsorption of single-stranded RNA in the presence of Mg^2+^ ions allowed for the selective removal of dsRNA contaminants from mRNA preparations obtained via in vitro transcription (IVT) [40]. This method enabled the removal of at least 80% of dsRNA while maintaining an mRNA recovery rate of approximately 85% [40]. A representative example of a carbon-based adsorbent developed for selective RNA purification is presented in Figure 3.

Key advantages of graphene oxide include rapid RNA capture and the so-called “shield effect,” which physically blocks access by RNases, thereby stabilizing RNA at room temperature for up to four days. The use of magnetic graphene oxide (MGO) further simplifies and shortens the procedure for isolating nucleic acids directly from cell lysates, bacteria, or blood through the application of an external magnetic field [13].

A potential limitation of carbon-based materials is the strong binding of RNA to the sorbent surface, which, in certain systems, hinders controlled desorption [40]. Furthermore, in the case of magnetic carbon composites, the unmodified magnetic component (iron oxide nanoparticles) tends to agglomerate due to electrostatic and magnetic forces; this can reduce biocompatibility and limit the sorbent’s available active surface area [22].

Overall, carbon-based materials offer rapid adsorption kinetics, high specific surface areas, and effective protection of adsorbed RNA against enzymatic degradation. However, strong adsorbate–surface interactions may complicate controlled RNA desorption and regeneration, which may limit their practical applicability in processes requiring RNA recovery and repeated adsorbent reuse.

### 3.2. Metal–Organic Frameworks (MOFs)

MOFs are crystalline porous materials composed of inorganic nodes—metal ions or clusters—linked by organic ligands [43]. The deliberate selection of the metal, linker, framework geometry, and functional groups enables precise control over pore size, polarity, stability, and the accessibility of active sites [43,44]. In the bioanalysis of nucleic acids, the ability to readily synthesize magnetic, hierarchical, and nanoscale MOFs is also of key importance [22,45].

**Figure 4:**
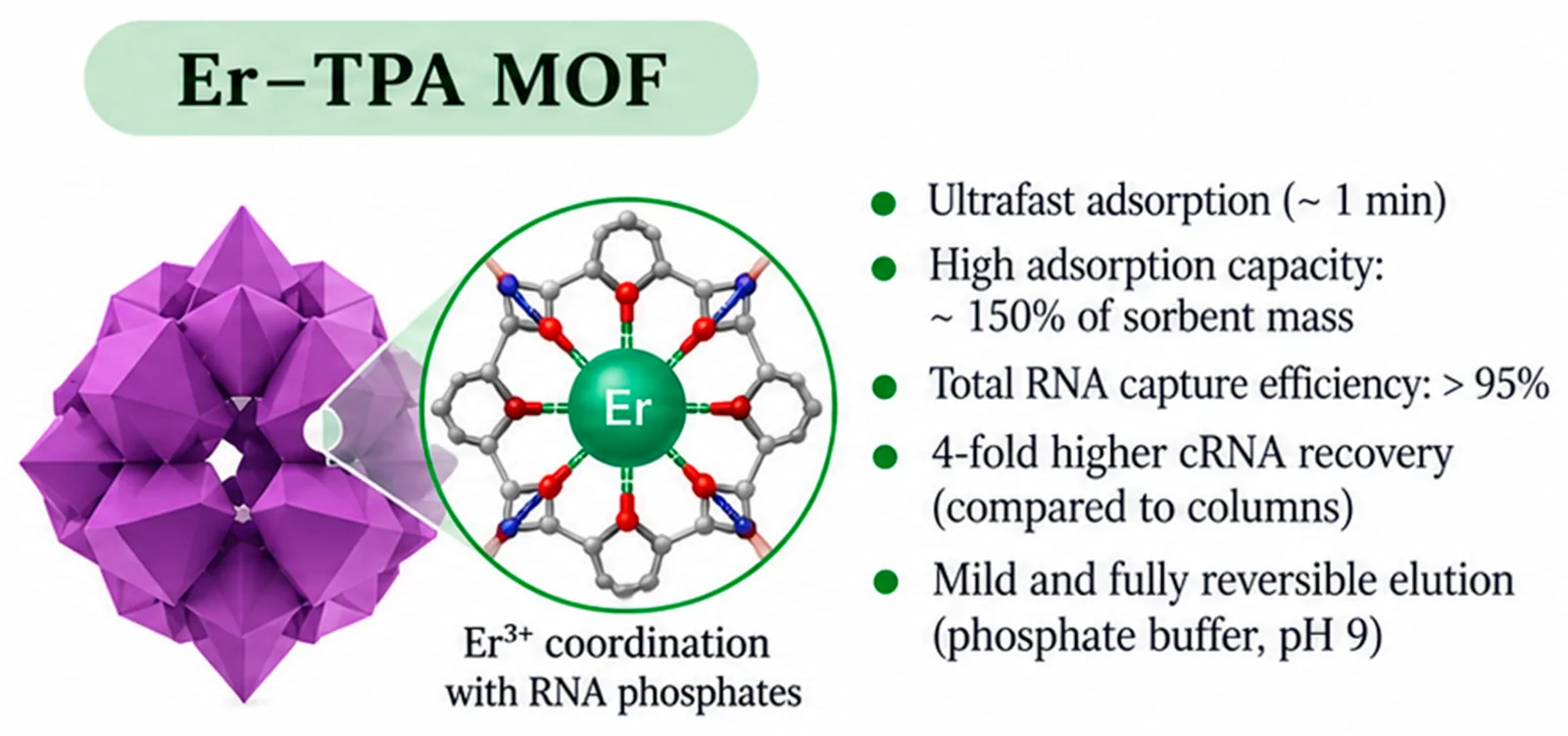
Er–TPA MOF enabling ultrafast, high-capacity RNA adsorption and reversible recovery.

In RNA adsorption processes, these frameworks can function as structurally programmable adsorbents, with the binding mechanism strictly determined by the specific framework structure. In the case of classical systems, such as mesoporous Co-IRMOF-74 frameworks, nucleic acid adsorption is purely physical in nature and relies on weak interactions, excluding classical electrostatic or coordination interactions [42]. In the case of advanced lanthanide-based frameworks (Ln-MOFs), the dominant mechanism is the strong coordination of hard Lewis acids (Ln^3+^) with the negatively charged phosphate backbone of RNA, further supported by zeta-potential-induced self-aggregation of the MOF particles [45]. These differences translate into unique application properties for both groups of materials. Studies on the Co-IRMOF-74 series have demonstrated that steric pore-size matching enables highly selective separation: the framework preferentially incorporates flexible single-stranded nucleic acids and structures with low thermal stability into its interior, while leaving rigid, stable conformations (e.g., G-quadruplexes) in solution [42]. Meanwhile, an optimized lanthanide-based Er–TPA MOF exhibits ultra-efficient, pore-size-independent nucleic acid capture (achieving an adsorption capacity of approximately 150% of the adsorbent’s mass within 1 min) and allows for their mild and fully reversible elution using a simple phosphate buffer [45]. A representative lanthanide-based MOF designed for rapid RNA capture and reversible recovery is shown in Figure 4. The material combines ultrafast adsorption kinetics with high capture efficiency and mild phosphate-buffer-mediated elution, illustrating the potential of MOF architectures for RNA isolation and monitoring applications [45].

Despite the potential for RNA selection based on size, conformation, and structural stability [42], the implementation of MOFs in routine analyses requires overcoming significant technological challenges. These primarily include the limited hydrolytic stability of many frameworks in aqueous environments, the risk of metal ion release (leaching), the need for thorough biocompatibility assessment, and challenges associated with material regeneration [22].

Compared with many other adsorbent classes, MOFs offer exceptional structural tunability and adsorption selectivity. Nevertheless, hydrolytic instability, potential metal-ion leaching, and regeneration challenges remain important limitations that may restrict their practical implementation in environmental systems.

### 3.3. Covalent Organic Frameworks (COFs)

**Figure 5:**
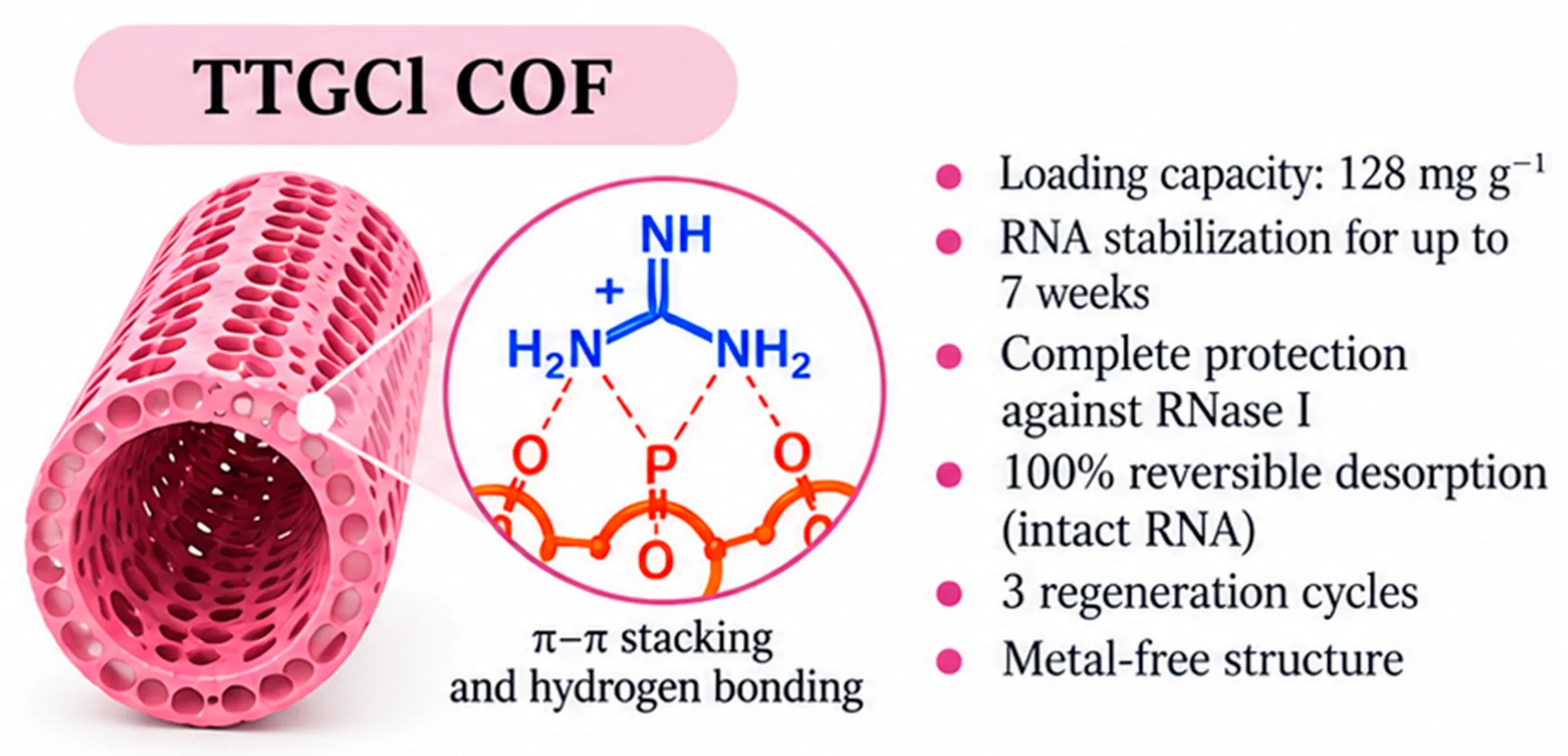
TTGCl covalent organic framework (COF) enabling RNA stabilization, protection against RNase-mediated degradation, and reversible recovery of intact RNA.

COFs constitute a class of crystalline organic polymers characterized by low framework density, high specific surface area, and precisely engineered pore geometry [46]. As metal-free materials, COFs eliminate the risk of transition metal ion release into bioanalytical systems, offering a significant advantage over conventional MOFs when handling delicate biological molecules [16]. Regarding nucleic acids, COF interactions rely on reversible non-covalent forces: electrostatic association with the negatively charged phosphate backbone, hydrogen bonding, and π–π interactions with nitrogenous bases. These specific physicochemical properties enable the use of COFs as functional nanoreservoirs for the controlled storage and protection of RNA at room temperature. A cationic framework with a nanotubular morphology (TTGCl), featuring guanidinium moieties, demonstrates exceptional efficiency. Spontaneous and exothermic RNA adsorption within TTGCl channels is driven by the synergy of electrostatic attraction and a dense network of hydrogen bonds with nitrogenous bases, a mechanism confirmed by isothermal titration calorimetry (ITC) and DFT calculations. The physical entrapment of RNA molecules within COF pores provides a protective shield effect, sterically hindering access by ribonucleases and preventing the enzymatic degradation of the nucleic acid for weeks under ambient conditions. This process is fully reversible; the desorbed RNA retains its structural integrity and biological activity, enabling its direct use as a template in RT-PCR reactions and sequencing [16]. A representative example of a COF designed for RNA stabilization and reversible recovery is shown in Figure 5. The TTGCl framework combines high RNA loading capacity with long-term stabilization, complete RNase protection, and recovery of structurally intact RNA, demonstrating the potential of COFs as multifunctional platforms for RNA storage, purification, and analysis [16].

Key technological barriers limiting the widespread implementation of COFs include difficulties in scaling up synthesis and the low hydrolytic stability of certain bonds in aqueous environments [46].

COFs combine high porosity with metal-free frameworks and frequently enable reversible RNA adsorption and recovery. However, their practical application is currently limited primarily by synthesis complexity, production costs, and the aqueous stability of certain framework chemistries.

### 3.4. Porous Polymers

**Figure 6:**
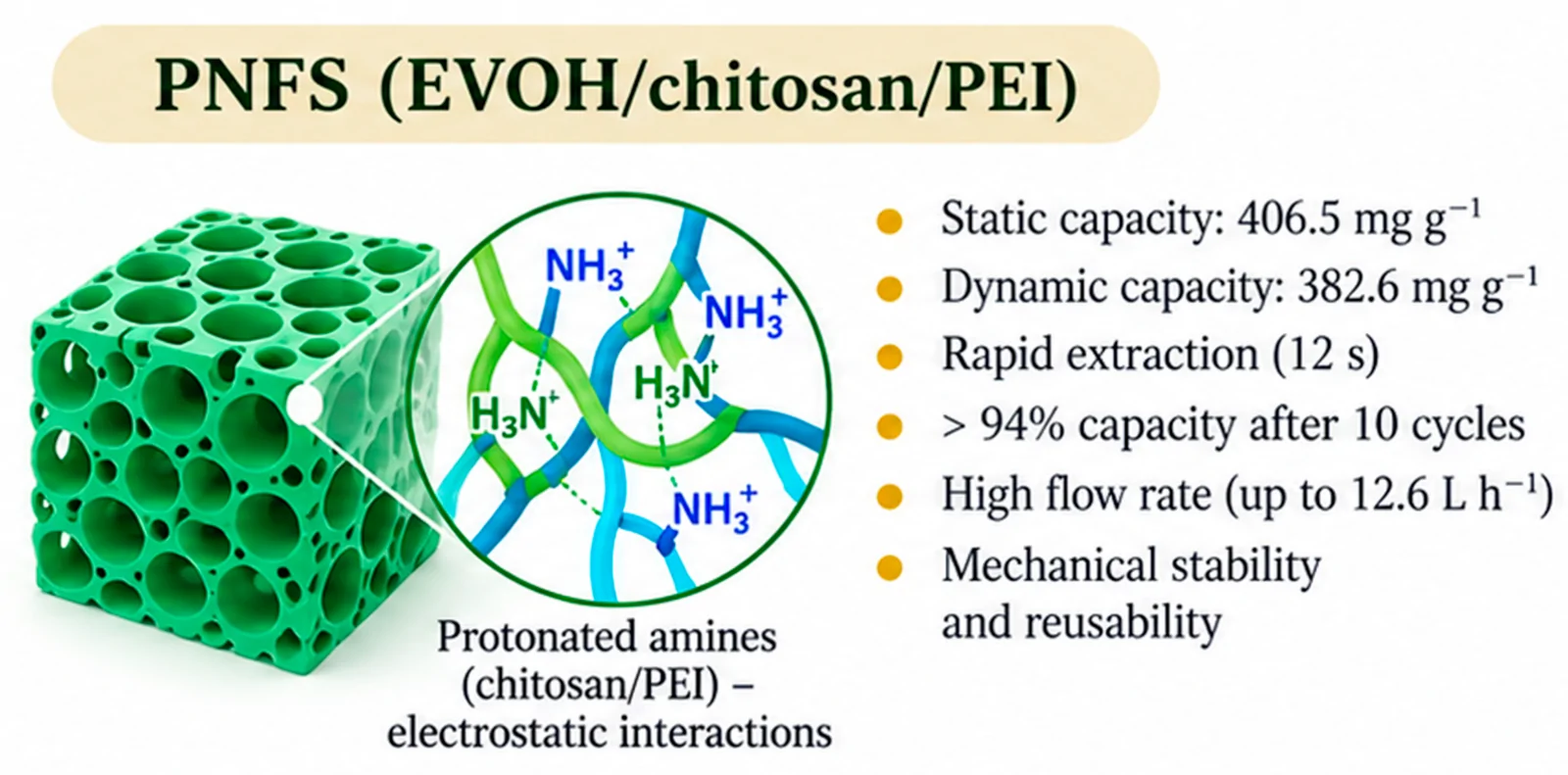
Porous nanofibrous sponge (PNFS) based on EVOH/chitosan/PEI for high-capacity RNA adsorption and repeated regeneration.

Porous polymers (including ion-exchange resins, cryogels, monoliths, and membranes) are characterized by excellent processability, allowing them to be easily formed into chromatographic beds—unlike brittle crystalline frameworks (MOFs, COFs) [46]. Their structural properties, such as charge, hydrophilicity, or porosity, can be precisely tuned [22]. The primary mechanism of RNA binding involves electrostatic interactions between phosphate groups and the polymer’s cationic centers (e.g., the amino groups of chitosan or PEI) [47]. Selectivity in separating RNA from crude biomolecule mixtures and cell lysates is enhanced through precise control of pore size (molecular sieving effect) or the use of boronate affinity interactions [22]. Hydrophilic, super-elastic nanofibrous sponges (PNFS) serve as an example of efficient 3D supports, enabling the rapid isolation of intact RNA [47]. Limitations of these materials include nonspecific protein binding, incomplete elution, and lower conformational selectivity compared to the precisely engineered pores of crystal lattices [42]. A representative example of a porous polymer adsorbent designed for rapid RNA isolation is shown in Figure 6. The PNFS architecture combines high adsorption capacity, rapid extraction kinetics, excellent mass-transfer properties, and retention of adsorption performance after repeated adsorption-desorption cycles, highlighting the potential of porous polymers for scalable RNA separation processes [47].

Porous polymers are advantageous because of their processability, scalability, and ease of incorporation into separation systems. However, compared with highly ordered porous materials such as MOFs and COFs, they generally provide lower structural selectivity and may exhibit greater nonspecific adsorption.

### 3.5. Porous Biomaterials

Biomaterials derived from natural lignocellulosic biomass—rich in cellulose, hemicellulose, and lignin—are being extensively studied due to their biodegradability, low toxicity, lightweight nature, and potential to replace non-biodegradable petrochemical products [48]. These sorbents can be fabricated into hydrogels, aerogels, membranes, and three-dimensional porous scaffolds [49]. An eco-friendly approach to synthesizing highly porous lignocellulosic matrices (e.g., from corn cobs or willow wood) involves regenerating them from solutions in biodegradable choline butyrate using water. These sorbents exhibit excellent thermal stability and high adsorption efficiency in water purification processes [48].

Chitosan is a key material in the context of RNA adsorption. In an acidic environment, its amino groups undergo protonation, enabling strong electrostatic binding to the negatively charged phosphate backbone of RNA and the formation of stable polyelectrolyte complexes. However, this strong association hinders the efficient elution of the nucleic acid using conventional isolation methods (e.g., TRIzol, CTAB), drastically reducing recovery yields. This process is strongly pH-dependent: the use of alkaline buffers (pH ~9) allows for the deprotonation of chitosan’s amino groups, thereby eliminating electrostatic interactions and enabling the release of intact, high-purity total RNA [49].

Conversely, in advanced flow systems, soft organic phases prone to collapse—such as cellulose-based ion exchangers—must be immobilized on rigid, inert mechanical scaffolds (e.g., reticulated vitreous carbon, RVC) to permit operation under high pressure [50]. To endow inherently inert biopolymers (such as pure cellulose or agarose) with the ability to bind RNA efficiently and in a controlled manner, it is necessary to graft them with cationic polymers (e.g., PEI) or functionalize them with boronate affinity ligands (BAC) [22]. Practical application of these biosorbents requires precise control over parameters such as environmental pH, the biopolymer’s degree of deacetylation, bed swelling, and protection against nuclease activity [49].

Biomaterials offer important advantages related to sustainability, biocompatibility, and low environmental impact. Nevertheless, adsorption performance and regeneration efficiency are often strongly influenced by environmental conditions, particularly pH, which may complicate large-scale applications.

### 3.6. Hybrid and Composite Materials

Hybrid materials combine components with different tasks, in which one phase provides binding ligands and the other improves mechanical stability, facilitates phase separation or optimizes mass transport [22]. Examples include the deposition of crystal lattices (MOF/COF) on polymer or magnetic supports, as well as cellulose-carbon composites and nanofibrous polymer sponges [47,50]. The dominant sorption interactions in these systems are multifunctional and based on the synergy of electrostatic forces, hydrogen bonds, π–π stacking interactions and the effect of spatial confinement of molecules in pores [43].

In flow systems, hybridization allows for a unique coupling of high chemical affinity with the mechanical resistance of the bed. An innovative solution is a composite combining a flexible cellulose anion exchanger with a rigid scaffold made of reticulated glassy carbon (RVC), which prevents the mechanical collapse of the organic phase under the influence of working pressure. This enables dynamic sorption of nucleic acids at extremely high flow rates, and microscopic analyses showed deep penetration and dense binding of biomolecules inside the highly porous structure of the flexible cellulose sorbent itself (Cytopore) [50]. Another advanced adsorbent is a superelastic nanofibrous sponge based on EVOH copolymer modified with chitosan and polyethyleneimine (PEI). Its oriented, macroporous structure ensures excellent mass transport, while the high density of cationic functional groups allows for rapid and highly selective isolation of intact RNA from mixtures containing proteins, lipids (lecithin) and DNA [47].

Despite good application properties, the complex hybrid architecture poses serious technological challenges [46]. These include primarily the need to ensure structural homogeneity, the risk of leaching of active ingredients, problematic regeneration and difficulties in maintaining the repeatability of serial production [22,46].

Hybrid materials often combine the advantages of multiple adsorbent classes, enabling improved adsorption efficiency, selectivity, and mechanical stability. However, the increased complexity of these systems may create challenges related to material homogeneity, regeneration, reproducibility, and large-scale manufacturing.

### 3.7. Comparative Assessment and Application Trade-Offs of Porous Materials for RNA Adsorption

To facilitate a critical comparison of the six classes of advanced porous materials discussed in Section 3.1, Section 3.2, Section 3.3, Section 3.4, Section 3.5 and Section 3.6, Table 1 summarizes their structural characteristics, dominant RNA-binding mechanisms, adsorption performance, recovery strategies, and regeneration potential. The table correlates physicochemical properties and pore architecture with reported adsorption capacities, experimental conditions, RNA desorption behaviour, and reusability. Particular attention is given to the practical trade-offs between adsorption efficiency, conformational selectivity, material stability, regeneration capability, and the recovery of structurally intact RNA. This comparative assessment highlights both the strengths and limitations of current adsorbent classes and identifies key knowledge gaps relevant to their large-scale implementation in environmental remediation and biosafety applications.

Comparison of the reported adsorption performance reveals substantial differences among the investigated adsorbent classes. Among the materials summarized in Table 1, porous polymers such as PNFS exhibit the highest reported adsorption capacities, reaching 406.5 mg g^−1^ under optimized conditions and retaining more than 94% of their adsorption capacity after 10 adsorption-desorption cycles [47]. Among crystalline porous materials, Er–TPA MOFs demonstrate exceptionally rapid adsorption kinetics and adsorption capacities approaching 150 wt.% [45], whereas TTGCl COFs provide effective RNA stabilization combined with complete and reversible RNA recovery and demonstrated reusability [16]. Carbon-based materials generally offer efficient RNA capture and protection against degradation, although strong adsorbate-surface interactions may hinder controlled desorption and regeneration [13,40]. In contrast, Co-IRMOF-74 frameworks provide unique conformational selectivity toward specific RNA structures but exhibit relatively low adsorption capacities and require destructive recovery procedures [42]. These observations indicate that no single adsorbent simultaneously maximizes adsorption capacity, selectivity, RNA recovery, regeneration potential, and operational simplicity. Consequently, material selection should be guided by the intended application, whether focused on RNA purification, environmental monitoring, wastewater treatment, or biosafety-related RNA capture and immobilization [13,16,40,42,45,47,49,50].

## 4. Mechanisms of RNA Adsorption

**Figure 7:**
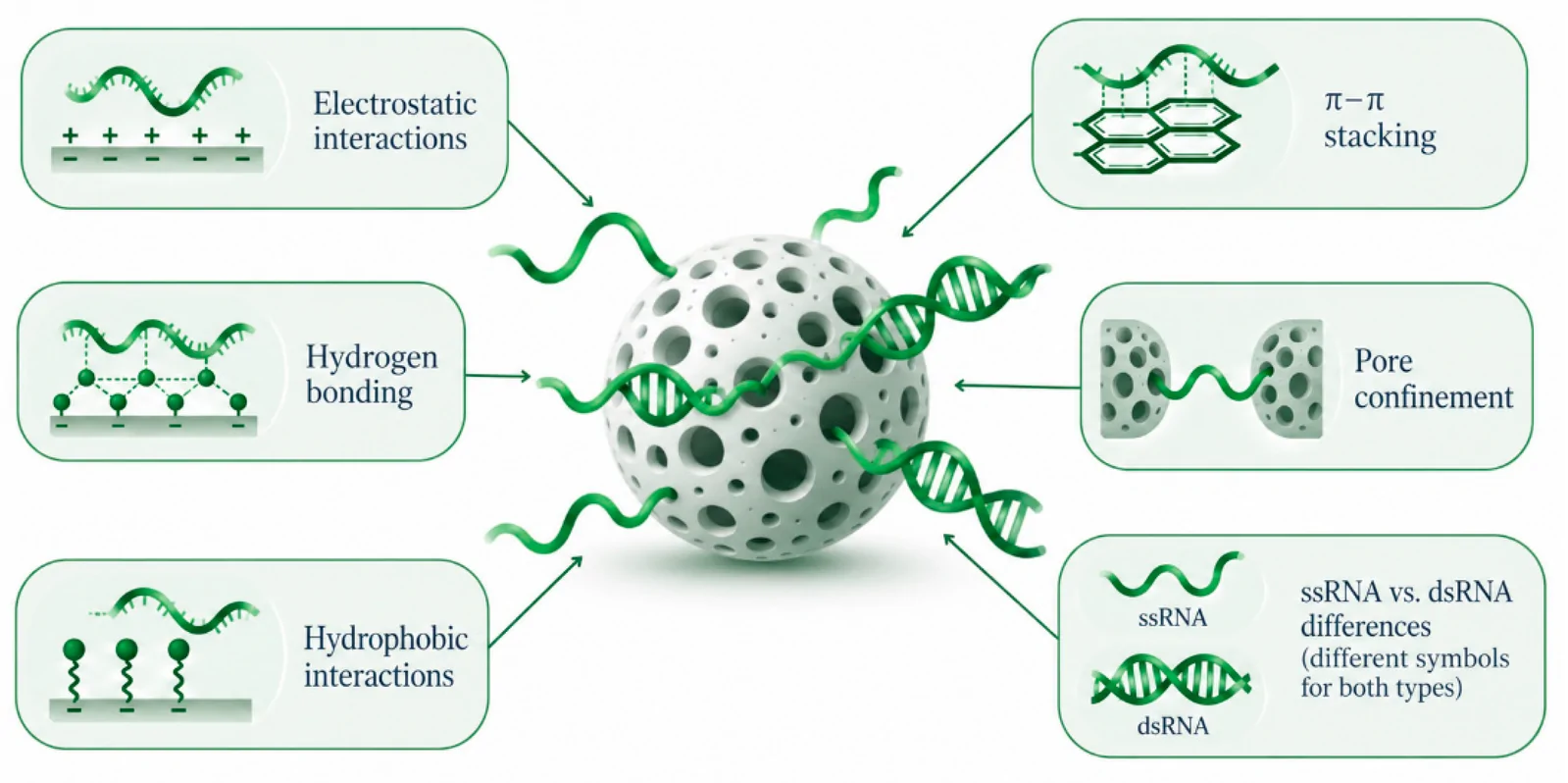
Schematic illustration of the major mechanisms governing RNA adsorption on advanced porous materials.

RNA adsorption on advanced porous materials (mesoporous silicas, MOFs, COFs, carbon nanomaterials, and modified zeolites) plays a key role in environmental remediation and biosafety. This process enables the effective capture and immobilization of RNA species, but may also contribute to their stabilization on material surfaces or within pore structures, potentially creating a risk of secondary release (desorption) under changing environmental conditions. The interaction of RNA with porous adsorbents is governed by a combination of electrostatic attraction, hydrogen bonding, cation bridging, π–π interactions, hydrophobic effects, and pore confinement. In biosafety-related applications, adsorption may reduce the mobility and environmental dissemination of RNA-containing particles; however, adsorption alone does not necessarily imply RNA degradation, irreversible inactivation, or loss of infectivity. Consequently, understanding the mechanisms governing RNA adsorption and desorption is essential for designing materials that effectively capture RNA while minimizing potential environmental and epidemiological risks [10,51]. The principal interactions involved in RNA adsorption and the influence of RNA structural features on adsorption behaviour are summarized schematically in Figure 7.

### 4.1. Electrostatic Interactions

The dominant mechanism of RNA adsorption on advanced porous materials is the electrostatic interaction between the negatively charged phosphate backbone of RNA and surfaces with a net positive charge. Materials such as amino-functionalized mesoporous silicas (MSN-NH_2_), cationic MOFs, and modified zeolites possess protonable groups (e.g., –NH_3_^+^), which strongly attract polyanionic RNA within the appropriate pH range. The strength of this interaction is strongly modulated by three main factors: solution pH—at lower pH (usually 4–6), protonation of surface groups increases, enhancing adsorption; ionic strength—higher salt concentrations (e.g., NaCl) screen electrostatic interactions, reducing adsorption capacity, although moderate salt concentrations can sometimes promote adsorption via the “salting-out” effect; the surface charge of the adsorbents (measured as zeta potential)—the higher the positive potential, the greater the binding efficiency. Additionally, divalent cations (Mg^2+^, Ca^2+^) play a significant role, acting as cation bridges that link the negatively charged phosphate groups of RNA to the adsorbent surface or stabilizing the secondary structure of the RNA itself, facilitating its adsorption under conditions similar to environmental or physiological conditions [12,52,53,54,55]. In the context of biosafety, electrostatic interactions enable rapid and effective capture and immobilization of RNA on porous surfaces. However, immobilization alone does not necessarily imply RNA degradation, loss of biological activity, or pathogen inactivation, as adsorbed RNA may remain structurally intact and potentially undergo desorption under changing environmental conditions. This mechanism is also reversible—changes in pH (e.g., alkalization) or an increase in ionic strength can lead to desorption and secondary release of RNA, which poses a potential transmission risk in plumbing, water filters, or laboratory waste. Therefore, the design of materials with a higher density of cationic groups or hybrid systems (electrostatics + covalent bonding) is crucial to minimize this risk [51,56].

### 4.2. Hydrogen Bonding and Coordination

Hydrogen bonds and coordination interactions constitute an important, complementary mechanism for RNA adsorption on advanced porous materials. Hydrogen bonds form between phosphate groups (PO_4_^3−^), ribose hydroxyls (2′-OH and 3′-OH), and nitrogen and oxygen atoms in the nitrogenous bases of RNA and functional groups on the adsorbent surface, such as silanol (–Si–OH), amine (–NH_2_), or carboxyl (–COOH) groups in mesoporous silicas, MOFs, or modified polymers.

In materials containing transition metal ions (e.g., Zr^4+^, Fe^3+^, Zn^2+^, Ti^4+^ in metal–organic networks), coordination also occurs, resulting in the formation of stable chelate complexes. These interactions are particularly strong in hydrated pores, where water can act as a hydrogen bridge, and exhibit a clear synergy with electrostatic interactions, increasing the overall binding energy. The strength of these mechanisms depends on the degree of surface hydration, pH, and the presence of donor–acceptor groups [12,57,58,59,60].

In the context of biosafety, hydrogen bonds and coordination significantly increase the stability of adsorbed RNA against enzymatic and chemical hydrolysis, protecting the molecules from degradation. Simultaneously, multipoint interactions can induce conformational changes (e.g., chain unwinding or destabilization of secondary structures), thereby altering its accessibility to enzymes and other biomolecules. However, the effects of such changes on RNA biological activity or pathogen infectivity require direct experimental verification and cannot be inferred solely from adsorption behaviour. This mechanism is particularly valuable in systems where permanent immobilization without complete desorption is desired [51,56].

### 4.3. π–π Stacking and Hydrophobic Interactions

π–π stacking and hydrophobic interactions play a key role in RNA adsorption on carbon-based materials such as graphene, carbon nanotubes (CNTs), activated carbons, as well as on aromatic linkers in MOFs and COFs. The π–π stacking mechanism involves the parallel stacking of aromatic rings of RNA bases, including purines (adenine A, guanine G) and pyrimidines (cytosine C, uracil U) with the π-electron surface of the adsorbents. Purines exhibit significantly stronger affinity due to their larger aromatic surface area and greater π-orbital overlap. Hydrophobic interactions complement this process, pushing nonpolar RNA fragments from the aqueous phase onto the hydrophobic porous surface. This mechanism is particularly important in materials with low surface polarity, where van der Waals forces and the hydrophobic effect dominate. Unlike electrostatic interactions, π–π stacking and hydrophobic interactions are relatively resistant to pH changes and moderate ionic strength fluctuations [53,57,61,62]. In the context of biosafety, these mechanisms ensure strong and often partially irreversible RNA binding, minimizing the risk of spontaneous desorption. At the same time, under environmental conditions, these interactions may contribute to the long-term stabilization and persistence of RNA associated with microplastic or carbon particles. However, RNA persistence should not be directly equated with infectivity or transmission potential, as these properties require independent biological assessment [51,63].

### 4.4. Pore Confinement Effects

Pore confinement (nanopore confinement) is one of the most characteristic mechanisms of RNA adsorption in advanced mesoporous materials. Pores with a diameter of 2–50 nm significantly increase the local RNA concentration, create multiple points of contact with the inner pore surface, and restrict the free diffusion of molecules. When the pore diameter is close to the dimensions of RNA (several to several dozen nanometers), an entrapment effect occurs, leading to unwinding of the poly-nucleotide chain, stabilization or destabilization of secondary structures, and impedance of access by enzymes (RNases) and substrates. The strength of this mechanism depends on the ratio of pore size to length and RNA conformation, the degree of functionalization of the inner pore walls, and the presence of solvents. In amino-functionalized mesoporous silicas, dsRNA conflation results in stronger binding compared to adsorption on the external surface, as confirmed by thermodynamic and spectroscopic studies [12,52,57,59,64].

In the context of biosafety, the pore confinement effect plays a particularly important role. Confining RNA within pores may reduce its mobility, alter its accessibility to enzymes, and influence its structural stability. However, pore confinement alone should not be interpreted as direct evidence of inhibition of replication, translation, or infectivity, as these outcomes require independent biological verification. Therefore, designing optimally sized pores and combining conflation with inactivation mechanisms (e.g., photocatalytic or chemical) is crucial to minimizing the risk of secondary release and transmission of pathogens [51,56,59].

### 4.5. Competitive Adsorption

In real environmental and biological matrices, RNA adsorption occurs under conditions of strong competition for active sites on adsorbent surfaces. RNA competes primarily with dissolved organic matter, proteins, microplastics, metal ions, and other nucleic acids (DNA, RNA fragments). DOM and proteins (e.g., albumins, globulins) often exhibit higher affinity for porous surfaces, thereby displacing RNA from adsorption sites. Microplastics, on the other hand, can act as a synergistic carrier, adsorbing RNA to their surface and facilitating its transport in aqueous environments. Metal ions (e.g., Fe^3+^, Cu^2+^, Ca^2+^) modify the charge and surface chemistry of adsorbents, simultaneously competing for coordination sites or forming ionic bridges [53,54,63,65].

The composition of the environmental matrix (municipal sewage, surface water, sediments)—including pH, ionic strength, humic substance content, and the presence of colloids—significantly reduces the efficiency of selective RNA adsorption. In multicomponent systems, isotherm models (Langmuir, Freundlich) require multicomponent extensions to reliably describe competitive processes. RNA adsorption kinetics are often slower in the presence of higher-affinity interferents [53,63,65].

### 4.6. Biosafety Implications of RNA Adsorption

Adsorption to advanced porous materials plays a crucial role in the capture and immobilization of pathogen-associated RNA. Depending on adsorbent properties and environmental conditions, adsorption may contribute to RNA stabilization, degradation, or facilitate subsequent inactivation processes. However, adsorption alone should not be considered direct evidence of pathogen inactivation or loss of infectivity. Adsorption mechanisms support inactivation through a complex combination of processes: denaturation of secondary structure (pore confinement and π–π stacking), physical immobilization of molecules that may limit their mobility and accessibility to biological systems (electrostatic interactions and hydrogen bonding), and facilitated access to chemical inactivation agents (e.g., guanidine solutions, detergents, or oxidants). Stabilization of RNA on surfaces or within pores alters its environmental mobility and persistence; however, stabilization alone does not demonstrate reduced infectivity or biological inactivation. Nevertheless, effective control of desorption processes remains necessary—for example, by introducing covalent bonds, barrier layers, or surface modifications that prevent RNA release under the influence of changes in pH, ionic strength, or the presence of competing adsorbates [51,56,64,66].

In the context of biosafety, hybrid materials combining adsorption capacity with antibacterial and antiviral properties, such as photocatalysis (e.g., TiO_2_), the release of metal ions (Ag^+^, Cu^2+^), or the generation of reactive oxygen species, are particularly promising. Such solutions may enable the simultaneous immobilization of pathogen-associated RNA and promote pathogen inactivation through additional mechanisms such as photocatalysis, metal-ion-mediated damage, or reactive oxygen species generation. In these systems, biological inactivation is supported not only by adsorption but also by independent destructive processes acting on the pathogen or its genetic material [51,56,66].

In summary, the multi-mechanistic adsorption of RNA on advanced porous materials forms the basis of sustainable remediation and biosafety technologies. Future research should focus on the dynamics of desorption, improving selectivity in complex environmental matrices, and integrating adsorption processes with effective inactivation methods to minimize the risk of secondary release of pathogenic RNA. It is important to distinguish between RNA capture, adsorption, immobilization, stabilization, degradation, and biological inactivation, as these endpoints describe different processes and should not be used interchangeably when assessing biosafety implications.

## 5. Analytical Methods for Studying RNA Adsorption

Understanding and optimizing RNA adsorption processes on advanced porous materials requires the use of a highly specialized set of analytical methods. These tools are essential not only for precisely determining the kinetics and efficiency of nucleic acid capture, but above all for verifying RNA integrity, degradation, and pathogen inactivation, which is the foundation of biosafety procedures [67,68,69].

The core research arsenal falls into three main categories: physicochemical surface analysis techniques that elucidate thermodynamics and the nature of bonds [70,71], quantitative and structural molecular biology methods, including advanced sequencing and amplification [72,73,74,75,76], and specialized spatial imaging and viability assays [77,78,79]. The choice of the optimal analytical route is always a compromise determined by the specific environmental matrix like material density and the presence of inhibitors, the desired sensitivity level, and the primary research goal—from simple confirmation of the physical retention of the biopolymer to rigorous proof of complete loss of the infectious potential of trapped pathogens.

### 5.1. Physicochemical and Thermodynamic Profiling of RNA Adsorption

While molecular biology methods address the integrity and infectivity of the trapped pathogen, understanding the mechanism of interaction between nucleic acids and porous surfaces requires the use of classical materials science tools.

To precisely determine which functional groups of a material participate in RNA capture, FTIR and X-ray photoelectron spectroscopy (XPS) are commonly used. These techniques allow for the unambiguous identification of hydrogen bonds and electrostatic interactions, as demonstrated, among other things, by tracing the associations of phosphate and amine groups of nucleic acids with charged surfaces. FTIR analysis is also crucial for confirming successful carrier modification, revealing the interactions of the material’s functional groups with the negatively charged RNA backbone [80,81].

Rational design of biosanitary materials requires determining the maximum adsorption capacity. Langmuir and Freundlich adsorption isotherm models are used for this purpose. These isotherms are widely used to describe equilibrium adsorption behavior and may provide insight into adsorption characteristics. Li et al. [82] reported that RNA adsorption often showed a better fit to the Langmuir model, whereas DNA adsorption was more appropriately described by the Freundlich model. However, such model fitting should be interpreted with caution. Agreement with the Langmuir equation may indicate adsorption behavior consistent with Langmuir assumptions but does not independently demonstrate monolayer adsorption or surface homogeneity. Similarly, fitting to the Freundlich model reflects adsorption on energetically heterogeneous surfaces but does not by itself establish a specific molecular adsorption mechanism. Therefore, mechanistic interpretations should be supported by complementary physicochemical and spectroscopic evidence. Similarly, for small RNA molecules (e.g., siRNA), adsorption behavior consistent with Freundlich-type models has been reported [81,82,83].

The time required to neutralize a threat is a key parameter of filtering systems. Fitting experimental data to mathematical models frequently indicates that nucleic acid adsorption on structured materials is well described by the pseudo-second-order kinetic model [81,83]. However, good agreement with the pseudo-second-order equation alone cannot be considered conclusive evidence of chemisorption or a specific adsorption mechanism. Rather, it may indicate that adsorption is influenced by surface-controlled processes. Mechanistic assignments should therefore be supported by complementary thermodynamic, spectroscopic, and structural analyses capable of identifying the nature of adsorbate–surface interactions.

Overall, adsorption isotherms and kinetic models are valuable tools for comparing adsorption systems and quantifying RNA removal performance. Nevertheless, neither equilibrium nor kinetic model fitting should be interpreted as direct proof of a specific adsorption mechanism. A comprehensive understanding of RNA adsorption requires integrating adsorption modeling with structural, spectroscopic, and thermodynamic characterization of both the adsorbent and the adsorbed phase.

### 5.2. Molecular Assessment of RNA Integrity and Inactivation

The pre-analytical stage—comprising extraction, filtration, and isolation—is of critical importance; improper execution of these processes—for example, failure to eliminate so-called legacy eDNA through rigorous DNase treatment—leads to false-positive results and erroneous assessments of sanitary status [6,67,84]. Moreover, the standard RT-qPCR method often targets short nucleic acid fragments, thereby failing to distinguish between fully infectious pathogens and those that have undergone physical fragmentation or inactivation [68]. To accurately assess the destructive impact of the adsorbent on RNA, the use of methods such as long-amplicon RT-qPCR (LA-qPCR) and the differential amplicon amplification (Delta Amp) method is recommended [72,73,74]. A significant decrease in long-fragment amplification efficiency serves as molecular evidence of physical damage to RNA caused by the porous material [72,74]. Furthermore, digital PCR (dPCR) enables highly sensitive, absolute quantification that is robust against environmental inhibitors [68], while propidium monoazide (PMA) allows for blocking the amplification of genetic material from damaged virions [75]. As part of a comprehensive assessment, direct RNA sequencing (e.g., using nanopore technology) avoids errors associated with reverse transcription and enables the mapping of structural degradation and epitranscriptomic modifications that influence RNA stability on engineered surfaces [76].

### 5.3. Spatial Imaging and Real-Time Biosensing

Environmental RNA found in complex matrices (such as biofilms on adsorbents) often adopts highly stable non-canonical forms (e.g., G-quadruplexes); detecting these requires targeted immunological labeling rather than standard dyes [77,79]. To understand the spatial distribution of entrapped pathogens, confocal laser scanning microscopy (CLSM) is combined with single-molecule fluorescence in situ hybridization (smFISH), enabling precise three-dimensional (3D) localization and quantification of individual RNA transcripts within the material’s pore structure [85]. Meanwhile, translating laboratory-based adsorption studies into environmental monitoring applications depends on the development of amplification-free biosensors. Integrating specific nucleic acid cleavage via CRISPR-Cas systems with microfluidic techniques, amphiphilic probes, or MOF-based nanozymes enables rapid, visual point-of-care detection [71,86,87]. Similarly, low-cost electrochemical sensors based on printed circuit boards show potential for the direct conversion of the adsorption process into a measurable electrical signal, which could find application in routine biosafety monitoring [88]. To facilitate the selection of appropriate verification tools for newly designed porous materials, the key techniques discussed in this chapter are summarized in Table 2.

Ultimate success in designing innovative porous materials depends on the holistic integration of surface chemistry with the above tools. Only such an approach can unequivocally demonstrate not only physical retention but, above all, the effective neutralization of biological threats.

On the one hand, the transition to eRNA analytics is biologically important, as it allows a more reliable assessment of whether pathogens trapped in materials are still alive and pose a real threat. On the other hand, however, researchers face a formidable barrier of methodological challenges: from extreme molecular instability and false positives during extraction to the dominant background noise from biofilms and fading signal at low pathogen concentrations. As a result, there is currently no single, universal, and error-free method that solves all these challenges simultaneously. This forces engineers and biologists to constantly compromise and employ complex, multi-pronged approaches—combining advanced amplification, spatial imaging, and rigorous quality control—to ultimately prove the biosanitary effectiveness of modern materials.

## 6. Environmental and Biosafety Applications

### 6.1. Water Treatment and Wastewater Remediation

Overall, wastewater constitutes a heterogeneous biological mixture containing cells, organic particles, metabolites, microorganisms, and nucleic acids [89,90]. RNA present in wastewater originates from multiple sources and includes not only material of human origin but also microbial transcripts and the genomes of viruses infecting humans, animals, plants, fungi, and bacteria [91].

Metatranscriptomic analyses of municipal wastewater have revealed the presence of a diverse pool of bacterial and viral transcripts [92], while viral RNA analyses also enable the identification and tracking of SARS-CoV-2 variants in wastewater [93]. Notably, plant viruses also constitute an important component of the environmental RNA pool. Dozens of plant virus species have been detected in wastewater, and representatives of the Virgaviridae may remain present even in treated wastewater [94,95]. This indicates that, from a biological perspective, a wastewater treatment plant is not solely a system for removing organic matter and fecal microorganisms but also acts as a barrier limiting the transfer of genetic material from wastewater into the environment.

The technological process of wastewater treatment is therefore multistage. In a typical system, it includes the mechanical and physical removal of larger particles and suspended solids, sedimentation during preliminary and primary treatment, followed by biological treatment based, among others, on activated sludge, biofilms, or membrane bioreactors, and, in more advanced installations, tertiary treatment involving filtration, membrane processes, adsorption, and final disinfection using UV irradiation, chlorination, ozonation, or advanced oxidation processes [96]. Each of these stages may reduce the concentration of viral particles and the amount of viral genetic material. Sedimentation, filtration, and adsorption primarily lead to the physical removal or sequestration of viral particles through their transfer from the aqueous phase to sludge, biofilm, membranes, or adsorbents. Biological treatment additionally promotes the retention of some viruses in activated sludge and the degradation of genetic material [97]. Disinfection, in contrast, constitutes the principal final biosafety barrier, the primary purpose of which is virus inactivation, i.e., the loss of viral infectivity [98,99]. Inactivation may, but does not necessarily, involve complete degradation of the genome [100]. UV irradiation directly induces photochemical damage to nucleic acids, whereas chlorine and ozone may modify both capsid or envelope proteins and the viral genome. Consequently, a positive RT-qPCR result after treatment is not equivalent to the presence of infectious viral particles [100].

Biological treatment is of fundamental importance for reducing viral load and genetic material; however, it does not always result in the complete removal of detectable RNA. Serra-Compte et al. [101], analyzing wastewater treatment lines, demonstrated a gradual decrease in the frequency of SARS-CoV-2 RNA detection during treatment. Viral material remained detectable in 23.3% of samples after secondary treatment, whereas no positive results were obtained after the application of membrane bioreactors and chlorination. A similar relationship was observed in Nepal, where SARS-CoV-2 RNA was detected in 47% of treated wastewater samples originating from installations using biological processes [102].

Enteric viruses are particularly important from the perspective of water biosafety because they may be excreted in large quantities, exhibit considerable environmental stability, and are not always completely eliminated by conventional treatment. In municipal wastewater treatment plants in Italy, enterovirus and norovirus RNA was detected both in influents and in treated effluents [103]. Similarly, Campos et al. [104] demonstrated the presence of human noroviruses after successive treatment stages. On the other hand, the application of advanced technologies may significantly increase removal efficiency. In a full-scale membrane bioreactor, Simmons et al. [105] achieved reductions of approximately 5.1 log_10_ for enteroviruses and 3.9 log_10_ for norovirus GII, while also demonstrating the importance of viral association with the solid fraction of wastewater.

Importantly, the effectiveness of treatment technologies should be evaluated not only with respect to human viruses. Bačnik et al. [94] identified representatives of 47 plant virus species in wastewater treatment plant influents and effluents and demonstrated that some tobamoviruses retained infectivity after conventional wastewater treatment. In a broader environmental context, these observations extend the concept of biosafety beyond the classical protection of human health. Insufficient removal of infectious plant viruses may have implications for plant health, crop safety, and systems using reclaimed water for irrigation. This has practical significance because treated wastewater is often ultimately discharged into rivers lakes and forest catchments or reused in water reclamation systems. Tandukar et al. [102] detected SARS-CoV-2 RNA in river waters, while earlier studies on enteroviruses and noroviruses indicate that treated effluents may remain a source of viral genetic material and, in the case of some viruses, also infectious particles, for natural aquatic ecosystems [100,103]. From a biosafety perspective, it is therefore crucial to limit the release of infectious virions before wastewater is discharged into the receiving environment. Detection of their genetic material remains an important tool for assessing treatment efficiency. It should also be emphasized that distinguishing between the detection of a molecular biomarker and the presence of an infectious virus represents one of the fundamental challenges in assessing wastewater treatment efficiency and biological risk.

In this context, adsorption may represent a particularly promising mechanism supporting remediation because it enables the physical transfer of virions and their associated RNA from the aqueous phase onto the surface of a solid material.

### 6.2. Monitoring, Biosurveillance and Biosafety

Technological progress, the development of molecular methods, and the growing importance of environmental monitoring have led to the expansion of biological surveillance from the classical assessment of water quality to the analysis of wastewater. Of particular importance in this context is wastewater-based epidemiology (WBE), in which changes in the concentrations of specific RNA markers can be used to monitor the occurrence of pathogens and the dynamics of their spread within a population. At the same time, the usefulness of RNA in environmental monitoring extends beyond the direct detection of pathogens. Selected RNA viruses can serve as markers of fecal load, enabling assessment of the degree of anthropogenic contamination of water and facilitating interpretation of changes in the concentrations of other pathogens detected in wastewater.

A fundamental limitation of molecular pathogen monitoring, however, is the relatively low concentration of target RNA in large sample volumes and complex environmental matrices, which additionally contain inhibitors of enzymatic reactions and often highly fragmented genetic material [96]. In this context, adsorption serves a different function than in conventional remediation. Its purpose may be the controlled capture and concentration of viral material prior to subsequent molecular detection.

The significance of this mechanism is particularly evident in passive wastewater monitoring. In contrast to one-time sample collection, an adsorbent placed directly in the wastewater stream can accumulate viral material over a defined period, providing more integrated information on the variability of contamination. Habtewold et al. [106] demonstrated that electronegative membranes can effectively accumulate SARS-CoV-2 from wastewater, with a linear increase in signal observed for up to 48 h of exposure. Similar utility of sorptive materials, including electronegative filters and cellulose-based materials, has also been demonstrated in other passive sampling systems. Notably, the use of adsorbents may simplify monitoring in locations where the collection of multi-hour composite samples is technically difficult. This has practical relevance for monitoring small catchments, hospitals, and other facilities of particular epidemiological importance [106].

The development of nanomaterials and porous materials also enables the integration of nucleic acid capture with molecular recognition mechanisms. For example, Hadynski et al. [107] used a metal–organic framework as a biosensing platform designed to recognize a model nucleic acid sequence corresponding to a fragment of the SARS-CoV-2 genome. The system generated both a fluorescent signal and a color change and enabled discrimination between oligonucleotides containing a single nucleotide mismatch. However, it should be emphasized that the study was a proof-of-concept and did not constitute direct validation of viral RNA detection in a complex environmental matrix.

Importantly, the ability of a surface to adsorb a virus is not equivalent to its inactivation. In this context, the term inactivation refers to the loss of viral infectivity rather than merely its immobilization on a surface. From a biosafety perspective, capture-and-inactivate materials are therefore particularly desirable, as they first capture the viral particle and subsequently lead to the loss of its infectivity [108]. For example, Shen et al. [109] developed photosensitized nanofibrous membranes that captured 99.2% of aerosols containing MHV-A59, used as a coronavirus model, and, following light exposure, resulted in the inactivation of 97% of viral particles in droplets through the generation of singlet oxygen. Such an approach may find applications in air filters, personal protective equipment, hospitals, diagnostic laboratories, and other environments with an elevated risk of biological contamination.

Surface contamination control is particularly important in laboratories using nucleic acid amplification methods. Residual nucleic acids and amplification products may represent a source of analytical contamination and false-positive results, even when they do not constitute an infectious hazard. For example, Lv et al. [110] detected SARS-CoV-2 RNA on multiple laboratory surfaces and equipment using ddPCR, highlighting the importance of contamination-control strategies in molecular laboratories. Moreover, disinfectants designed primarily to inactivate microorganisms differ in their effectiveness in degrading free nucleic acids [111]. In this context, surfaces capable of adsorbing, immobilizing, or catalytically degrading RNA could in the future constitute an additional element of contamination control.

Overall, adsorption-based materials can support biosafety applications not only through RNA capture and concentration but also through biosensing, environmental monitoring, filtration, and contamination-control strategies.

### 6.3. Nature-Based Solutions

In a broader environmental context, the development of adsorption technologies should take into account not only the efficiency of RNA and viral particle capture, but also material cost, durability, and the potential environmental burden associated with production and disposal. Conventional synthetic adsorbents may be characterized by a high specific surface area and controlled surface properties; however, their production and subsequent disposal may increase the environmental cost of the remediation process. For this reason, increasing attention is being given to solutions based on biopolymers, naturally derived materials, and biomass converted into porous adsorbents [112,113]. Materials such as alginate, cellulose, and chitosan, as well as biomass-derived adsorbents such as biochar, are particularly interesting because of their renewable or waste-derived feedstocks, the possibility of surface modification, and their capacity to immobilize biological particles. It should, however, be emphasized that not all materials belonging to this group are biodegradable, particularly after chemical functionalization or in the case of biochar.

Alginate biocomposites provide a good example, in which the natural polysaccharide forms a porous matrix allowing the incorporation of an additional adsorbent. Gouthro et al. [114] combined sodium alginate with powdered activated carbon to obtain PA-NaA hydrogel beads capable of capturing viruses directly from wastewater. In kinetic experiments, the material enabled efficient capture of heat-inactivated SARS-CoV-2 after only a few minutes of contact, followed by its elution and RNA analysis using RT-qPCR. The applicability of the material was also confirmed through the detection of endogenous SARS-CoV-2 in wastewater samples. Importantly, this approach was designed as a simpler alternative to more time-consuming virus concentration methods [114].

Similar possibilities are offered by cellulose- and chitosan-based materials. Cationically modified nanocellulose effectively adsorbed the bacteriophages MS2 and Qβ, while changes in pH also enabled partial, controlled desorption of previously bound particles [115]. From a biological perspective, this is particularly interesting because the surface properties of the material can be adjusted to the charge of the viral capsid, allowing electrostatic interactions to be used as the principal separation mechanism. Chitosan, a natural polysaccharide obtained from chitin, can, after the introduction of positively charged quaternary groups, form nanofibrous materials capable of adsorbing both enveloped and non-enveloped viruses. Mi et al. [116] demonstrated that water-stabilized chitosan nanofibers retained their adsorption properties and indicated their potential use in low-pressure microfiltration membranes for water treatment.

Biochar occupies a particular position within this group because its developed porous structure, feedstock availability, and potential production from various types of biomass make it an attractive material for water and wastewater treatment systems [117]. In addition to agricultural residues, forest biomass may also serve as a valuable precursor for the production of porous carbon-based adsorbents intended for environmental remediation applications. Its sorption properties can be further enhanced through chemical surface modification. Sun et al. [118] used quaternized biochar in a constructed wetland system and demonstrated that the material not only enhanced virus adsorption but also caused destabilization of the capsid structure. These observations indicate the possibility of combining sorptive functionality with effects leading to a loss of viral particle integrity within a single material.

Notably, *Moringa oleifera* seed extract, containing water-soluble cationic proteins, has been used to develop a simple method for concentrating viruses from wastewater. The method was validated for both enveloped and non-enveloped viruses and subsequently used for the detection in wastewater of, among others, PMMoV, noroviruses, enteroviruses, influenza A virus, and SARS-CoV-2 [119]. Such an approach may reduce dependence on specialized membranes and costly infrastructure, thereby increasing the accessibility of virological monitoring under resource-limited conditions. Consequently, solutions based on naturally derived materials may find applications in both water and wastewater remediation, as well as in simple filtration systems and environmental *biosurveillance*.

In light of the above data, it is clear that combining natural matrices with controlled surface functionalization may lead to the development of materials integrating adsorption, concentration, filtration, and, in some cases, viral particle inactivation. A key direction for future research will therefore be the development of materials that, while retaining high affinity for the target biological material—RNA, viral components, or intact virions—remain inexpensive, environmentally safe, regenerable or biodegradable, depending on their nature, and suitable for use outside centralized laboratory infrastructure and large wastewater treatment plants. Such an approach may enable the development of low-cost biosafety systems tailored both to environmental protection and to limiting the biological risks associated with the transport of viruses and their genetic material.

### 6.4. RNA Desorption, Adsorbent Regeneration and Reusability

The practical implementation of adsorption-based technologies requires consideration not only of RNA capture efficiency but also of RNA desorption, adsorbent regeneration, material stability, and long-term reusability. These factors directly influence process sustainability, operational costs, and the feasibility of large-scale environmental applications [22,46].

Desorption of adsorbed RNA may occur naturally following changes in environmental conditions, such as pH, ionic strength, temperature, or the presence of competing ions and organic compounds. For example, RNA associated with mesoporous silica can be released through disruption of electrostatic interactions and hydrogen bonding in the presence of salts, EDTA, or urea [12]. Similar phenomena may occur in environmental systems where fluctuations in water chemistry modify adsorbent–RNA interactions. Therefore, RNA adsorption should not always be regarded as irreversible immobilization [12,49].

From an analytical perspective, controlled RNA desorption may be advantageous because it enables the recovery and isolation of intact RNA for downstream applications such as RT-qPCR, sequencing, environmental monitoring, and biosurveillance. Several porous materials, including selected MOFs and COFs, have demonstrated the ability to adsorb RNA efficiently while allowing mild elution and recovery of structurally intact molecules. Such properties are particularly valuable in monitoring applications where concentration and subsequent molecular analysis of environmental RNA are required [16,45].

Adsorbent regeneration is equally important for practical deployment. Regeneration strategies typically rely on pH adjustment, washing with salt solutions, chelating agents, organic solvents, or competing ligands capable of disrupting adsorbate–surface interactions [12,49]. However, repeated regeneration cycles may gradually reduce adsorption capacity due to pore blockage, loss of functional groups, material degradation, or incomplete desorption of retained biomolecules [22,46].

The regeneration potential varies considerably among adsorbent classes. Carbon-based materials often exhibit strong RNA binding, which may hinder complete regeneration [22,40]. MOFs and COFs can provide controlled adsorption and desorption, although framework stability under repeated cycling remains an important consideration [16,45,46]. Porous polymers and biopolymer-based adsorbents generally allow relatively simple regeneration procedures but may experience reduced selectivity or structural changes after multiple cycles [47,49].

Future studies should place greater emphasis on adsorption–desorption cycling experiments, long-term material stability, regeneration efficiency, and RNA recovery yields under realistic environmental conditions. Such information remains limited, but is essential for evaluating the practical and economic feasibility of RNA adsorption technologies in environmental remediation and biosafety applications [22,46].

## 7. Future Perspectives and Current Challenges

### 7.1. Current Challenges

Despite promising research results, the implementation of RNA adsorption technologies on advanced porous materials faces numerous scientific, technical, and regulatory challenges that limit their practical application in environmental remediation and biosafety systems.

The low chemical and enzymatic stability of RNA is one of the fundamental limitations. RNA molecules are highly susceptible to hydrolytic degradation and the action of ubiquitous RNases, which makes obtaining reproducible and reliable adsorption, kinetics, and isotherm measurement results significantly difficult, especially in long-term environmental studies. This requires the use of RNase inhibitors and strict temperature and pH control, complicating analytical procedures and increasing research costs [10,12].

Another major challenge is the complexity of the environmental matrix. In real-world samples such as municipal wastewater, surface water, or sediments, RNA occurs in the presence of a vast number of interfering agents—dissolved organic matter, proteins, humic substances, colloids, microplastics, and metal ions. These components compete strongly for adsorption sites, significantly reducing the selectivity and overall efficiency of the process. The lack of universal mathematical models describing adsorption in multicomponent systems further limits the ability to predict the behaviour of materials under real-world conditions [53,54,63,65].

The lack of standardization of research methods is another significant barrier. The wide variety of experimental protocols used—differences in adsorbent types, pH conditions, ionic strength, and RNA concentration determination techniques (qPCR, spectrophotometry, fluorescence)—makes it difficult to compare results between laboratories and conduct reliable meta-analyses [12,64].

Similarly, the number of systematic comparative studies testing different classes of materials (silicas, MOFs, carbon materials, hybrids) under identical conditions is limited, making it difficult to select the most effective adsorbents for specific applications [57,59].

Significant challenges also arise during process scale-up. Most research is conducted on a laboratory scale (milligrams to grams of adsorbents). Transitioning to pilot and industrial scales is associated with challenges related to the high cost of synthesizing some materials (especially MOFs and COFs), regeneration and reuse of adsorbents, column flow hydraulics, and decreased adsorption efficiency with larger volumes of processed solutions [59,64]. From a biosafety perspective, these challenges are particularly significant. Research on the adsorption of RNA from pathogenic viruses requires experiments to be conducted under BSL-3 or BSL-4 conditions, which limits the number of research centers capable of conducting such work and significantly extends the technology development timeline [56]. There is also a risk of secondary RNA release—stabilization of molecules on porous surfaces can lead to their long-term persistence, while sudden changes in conditions (pH, ionic strength, or the presence of surfactants) can cause desorption and potential transmission of genetic material or infectious RNA into the environment. The lack of uniform standards for validating pathogen RNA inactivation on adsorbents, certifying antiviral materials, and establishing procedures for the safe disposal of used adsorbents further complicates the implementation of these technologies in practice [51,56,66].

In summary, overcoming these challenges requires an interdisciplinary approach combining expertise in materials chemistry, molecular biology, process engineering, and biosafety. Key directions for future research include the development of standardized protocols, the design of selective and regenerable materials, the development of hybrid adsorption-inactivation systems, and the creation of a regulatory framework for the safe use of such materials in health and environmental protection. Addressing these challenges is essential for the successful development and implementation of next-generation RNA adsorption technologies. Several promising directions that may help overcome these limitations are discussed below.

### 7.2. Future Perspectives

The future of research on RNA adsorption on advanced porous materials is emerging as one of the most promising directions in the fields of environmental remediation, epidemiological monitoring, and biosecurity. Further technological development is expected to enable not only more effective removal of pathogen RNA but also the creation of intelligent, multifunctional systems with controlled properties.

A key area is the design of materials with precisely controlled surface chemistry. Advanced synthesis methods (e.g., postsynthetic modification, layered deposition) will enable the creation of adsorbents with optimized functional group density, hydrophilicity/hydrophobicity, and surface charge. Such materials should demonstrate higher selectivity for RNA even in the presence of strong interfering agents [120,121].

Another promising direction is the development of stimuli-responsive adsorbents. These materials can change their properties (e.g., release or bind RNA more strongly) in response to external signals such as changes in pH, temperature, light, or the presence of specific ions. This functionality will enable controlled adsorption, desorption, and regeneration of adsorbents, which are crucial for process economics and biosafety [122,123].

Integrating adsorption with other inactivation mechanisms, such as enzymatic degradation (immobilized RNases), photocatalysis, or the controlled release of metal ions, will enable the creation of hybrid “one-step” materials that simultaneously immobilize and irreversibly inactivate pathogen RNA. Applications in wastewater-based epidemiology (WBE) are particularly promising, as porous adsorbents can be used to effectively concentrate and stabilize viral RNA directly from large volumes of wastewater, significantly increasing the sensitivity and speed of pathogen detection [124]. Advances in molecular modeling (molecular dynamics simulations (MD), QM/MM methods) and machine learning will enable the precise prediction of RNA-surface interactions at the atomic level. This will make the design of new materials significantly faster and more rational [57,125].

In the area of biosecurity, the development of next-generation antiviral materials, combining high adsorption capacity with built-in inactivation mechanisms, will be particularly important. Adsorbents designed for rapid sample collection and stabilization in the field (point-of-care), as well as the integration of adsorption systems with biosensors and real-time diagnostic platforms, are also promising. Such solutions could become a component of global early warning systems for epidemic outbreaks [126,127,128].

In summary, the future development of RNA adsorption technology will require close interdisciplinary collaboration between chemists, biologists, engineers, and biosafety experts. Implementing these perspectives will not only enable effective environmental protection but also significantly enhance biosafety at a global scale.

## 8. Conclusions

Free nucleic acids in aquatic environments—whether natural (such as viral RNA and eRNA) or synthetic (such as the dsRNA used in biopesticides)—pose a growing challenge for environmental engineering and biosafety. Their persistence, susceptibility to degradation, and complex interactions with environmental matrices affect monitoring reliability and may impact non-target organisms, including pollinators. This review demonstrates that advanced porous materials hold great potential for RNA capture, with their efficacy depending primarily on pore architecture, surface charge, and the presence of functional groups. Adsorption mechanisms are multifaceted, involving electrostatic interactions, hydrogen bonding, hydrophobic and π–π interactions, and pore entrapment, with significant differences observed between ssRNA and dsRNA. Environmental conditions—such as pH, ionic strength, dissolved organic matter, metals, and competing molecules—significantly modulate both RNA sorption and desorption. Practical applications include the treatment of surface water, wastewater, and agricultural runoff, particularly regarding the management of dsRNA-based biopesticides and pollinator protection. Functionalized biochar stands out as a highly promising material, potentially serving as the basis for low-cost filtration barriers. At the same time, key limitations in current research are highlighted, including the lack of methodological standardization and the difficulty of translating laboratory findings to complex environmental matrices. In the future, it will be necessary to design selective, stable, and regenerable materials capable of the controlled capture, stabilization, or inactivation of RNA, as well as to develop analytical methods that enable a reliable assessment of the fate of RNA in real-world aquatic systems. Ultimately, the challenge extends beyond adsorption chemistry itself. Only by reimagining RNA-capturing materials as integral components of environmental biosafety infrastructure will they prove as reliable in the field as they have been in the laboratory.

## Figures and Tables

**Table 1:** Comparative summary of porous adsorbents for RNA adsorption, recovery, regeneration and reuse.

Adsorbent	RNA Type	Adsorption Performance	Experimental Conditions	RNA Recovery/Desorption	Regeneration/Reusability	Main Advantages	Main Limitations	Ref.
GO@PEG-PS	mRNA, dsRNA	≥80% dsRNA removal; ~85% mRNA recovery	Presence of Mg^2+^ ions	Mild recovery of target mRNA after purification	Not reported	Selective removal of dsRNA impurities; high recovery	Regeneration data unavailable	[40]
Magnetic graphene oxide (MGO)	Total RNA	Rapid RNA isolation from biological matrices	Magnetic separation	RNA recovered after isolation procedure	Not reported	Fast separation; simple operation	Potential nanoparticle aggregation; limited reuse data	[13]
Co-IRMOF-74	ssRNA, structured nucleic acids	Selective adsorption based on conformation and pore matching	Mesoporous MOF system	Selectivity based on molecular structure	Reusability not reported	High selectivity	Hydrolytic stability concerns	[42]
Er–TPA MOF	Nucleic acids	Adsorption capacity ≈ 150 wt.% within 1 min	Phosphate-buffer elution	Fully reversible elution of nucleic acids	Regeneration potential suggested but not systematically evaluated	Extremely rapid adsorption; efficient recovery	Framework stability and scale-up challenges	[45]
TTGCl COF	RNA	Efficient adsorption and protection of RNA	Ambient conditions	Reversible RNA release with preserved integrity	Demonstrated repeated loading/release capability	RNA stabilization; metal-free architecture	Complex synthesis; aqueous stability issues	[16]
Porous nanofibrous sponge (PNFS)	Total RNA	Rapid and efficient RNA isolation	Flow-through operation	Efficient RNA recovery	>94% adsorption capacity retained after 10 cycles	Excellent mass transfer; demonstrated reuse	Lower structural selectivity than crystalline frameworks	[47]
Chitosan-based adsorbents	Total RNA	Strong RNA capture via electrostatic interactions	pH-dependent adsorption	RNA release achieved at alkaline pH (~9)	Not systematically evaluated	Biocompatible; low cost	Strong binding may hinder recovery	[49]
Cytopore@RVC composite	Nucleic acids	High-capacity dynamic adsorption under flow conditions	High-flow chromatographic operation	Elution possible under chromatographic conditions	Operational stability demonstrated; long-term reusability not fully assessed	Suitable for large-scale separation systems	Complex architecture	[50]

**Table 2:** Summary of selected analytical methods in RNA adsorption and stability studies.

Method Category	Specific Techniques	Contribution to RNA Adsorption Studies	Key Limitations	Ref.
Physicochemical Characterization	FTIR, XPS, Adsorption Isotherms, Kinetic Models	Identifies specific binding sites (e.g., electrostatic, H-bonds) and estimates maximum adsorption capacity.	Mathematical fits (e.g., pseudo-second-order) do not independently prove molecular mechanisms (e.g., chemisorption).	[80,81,82,83]
Molecular & Integrity Assessment	LA-qPCR, dPCR, PMA-qPCR, Direct Nanopore Sequencing	Differentiates between physical entrapment and permanent RNA degradation; verifies pathogen inactivation.	Extraction from porous matrices can be highly inefficient; standard RT-qPCR may cause false positives by amplifying short, biologically inactive fragments.	[6,67,68,72,73,74,75,76,84]
Spatial Imaging	CLSM, smFISH, Specific Immunolabeling	Visualizes the 3D spatial distribution of RNA within material pores and surrounding biofilms.	High background noise in environmental matrices; complex optimization required for thick or recalcitrant biofilms.	[77,79,85]
Rapid Biosensing	CRISPR-Cas assays (e.g., Cas12a/13a), Electrochemical Sensors	Enables real-time, amplification-free detection of captured RNA for field biosafety applications.	Susceptible to environmental inhibitors released from the adsorption matrix; sensitivity drops at extremely low RNA concentrations.	[71,86,87,88]

## Data Availability

No new data were created or analyzed in this study. Data sharing is not applicable to this article.
